# Genetic programming of macrophages to perform anti-tumor functions using targeted mRNA nanocarriers

**DOI:** 10.1038/s41467-019-11911-5

**Published:** 2019-09-03

**Authors:** F. Zhang, N. N. Parayath, C. I. Ene, S. B. Stephan, A. L. Koehne, M. E. Coon, E. C. Holland, M. T. Stephan

**Affiliations:** 1 Clinical Research Division, Fred Hutchinson Cancer Research Center, Seattle, WA 98109 USA; 2 Department of Neurological Surgery, University of Washington School of Medicine, Seattle, WA 98195 USA; 3 Comparative Pathology, Fred Hutchinson Cancer Research Center, Seattle, WA 98109 USA; 4 Human Biology Division, Fred Hutchinson Cancer Research Center, Seattle, WA 98109 USA; 5 Alvord Brain Tumor Center, University of Washington, Seattle, WA 98195 USA; 6 Department of Bioengineering and Molecular Engineering & Sciences Institute, University of Washington, Seattle, WA 98105 USA; 7 Department of Medicine, Division of Medical Oncology, University of Washington, Seattle, WA 98195 USA

**Keywords:** Cancer immunotherapy, Nanobiotechnology

## Abstract

Tumor-associated macrophages (TAMs) usually express an M2 phenotype, which enables them to perform immunosuppressive and tumor-promoting functions. Reprogramming these TAMs toward an M1 phenotype could thwart their pro-cancer activities and unleash anti-tumor immunity, but efforts to accomplish this are nonspecific and elicit systemic inflammation. Here we describe a targeted nanocarrier that can deliver in vitro-transcribed mRNA encoding M1-polarizing transcription factors to reprogram TAMs without causing systemic toxicity. We demonstrate in models of ovarian cancer, melanoma, and glioblastoma that infusions of nanoparticles formulated with mRNAs encoding interferon regulatory factor 5 in combination with its activating kinase IKKβ reverse the immunosuppressive, tumor-supporting state of TAMs and reprogram them to a phenotype that induces anti-tumor immunity and promotes tumor regression. We further establish that these nanoreagents are safe for repeated dosing. Implemented in the clinic, this immunotherapy could enable physicians to obviate suppressive tumors while avoiding systemic treatments that disrupt immune homeostasis.

## Abstract (web summary)

The previous efforts to reprogramme tumour-associated macrophages (TAMs) to M1 phenotype have caused undesired side-effects. Here, the authors report targeted nanocarriers for delivering mRNA encoding M1-polarizing transcription factors to TAMs and show their efficacy in multiple mouse tumour models.

## Introduction

Tumor-associated macrophages (TAMs) are one of the most abundant non-neoplastic cell types found in discrete cancer locales^1^. Just like in healthy tissues, where macrophages have a remarkable ability for responding to environmental cues, TAMs are educated by the tumor microenvironment they experience. This produces multiple phenotypes that have a broad range of functions. TAM phenotypes can be described along a linear scale, where M1 and M2 phenotypes represent the two extremes (comparable to the T_H_1–T_H_2 classification)^2,3^. M1 macrophages are recognized as classically activated macrophages that can phagocytose pathogens. More importantly, these cells have anti-tumoral properties^4,5^. Unfortunately, other macrophages are polarized into the M2 phenotype. These decrease inflammation, encourage tissue repair, and provide pro-tumoral effects^6^. In established progressive tumors in humans, TAMs usually express an M2-like phenotype, and thus promote tumor progression, metastasis, and resistance to chemotherapy^7–9^. It is therefore of key interest in cancer research to create strategies that can reprogram TAMs from a pro-tumoral (M2-like) to an anti-tumor (M1-like) phenotype and thereby induce immune effects that can bring about tumor regression. However, as yet there are no methods that can enable physicians to rationally and selectively reprogram TAMs for therapeutic purposes^10^.

For example, interleukin-12 (IL-12), IFN-γ, Toll-like receptor (TLR-) agonists, and CD40 agonists have all been reported to induce repolarization of TAMs^11–13^. However, these immunomodulatory agents can activate a broad range of cell types, which means they are associated with dose-limiting adverse effects and systemic toxicities^14–16^. Likewise, several small molecule drugs have been developed that focus on blocking the localization of TAM-precursor cells to tumors by targeting pathways involved in cell recruitment or expansion (e.g., inhibitors of CSF-1/CSF-1R^17,18^ or CCL2^19^). Unfortunately, these approaches do not specifically promote macrophage anti-tumor activities and require repeated systemic exposure to large doses of the drugs. Furthermore, clinical trials of these pharmaceuticals produced low responses unless they were combined with cyto-reductive therapies or checkpoint blockade inhibitors^19,20^. Another factor that complicates the clinical use of CSF-1R inhibitors is the systemic depletion of normal monocytic cells, which causes high toxicity if patients are treated for prolonged periods^21^.

Taking advantage of the very efficient phagocytic uptake of particles by macrophages, conventional nanocarriers such as liposomes have been formulated with bisphosphonates or other anti-proliferative agents (e.g., liposomal-clodronate)^22^ as a means to systemically deplete these cells, which are replaced via natural regeneration mechanisms. Also, oncolytic viruses have been used to deliver siRNA to silence immune-evasion pathways within tumors and indirectly promote phagocytosis of TAMs^23^. However, neither of these approaches reprograms existing macrophages with tumor-fighting capabilities.

Macrophage mannose receptor 1 (MRC1), also known as CD206, is a type I transmembrane protein that belongs to the C-type lectin family. This receptor is expressed by macrophages and dendritic cells^24^. M2-like TAMs are derived from circulating monocytes that already express CD206, which is further upregulated upon extravasation of the cells at the tumor site and by exposure to factors in the perivascular tumor microenvironment^25^. Because CD206 shows high expression levels in TAMs, strategies that optimize the uptake of therapeutics via these receptors have the potential to be extremely powerful. Furthermore, this directed uptake is likely to require smaller treatment doses, thereby reducing the toxicity of the delivered substances. Huhn et al., for instance, functionalized polymer nanogels as potential drug nanocarriers with a Nanobody specific for CD206^26^, and similar concepts have been described by other groups to preferentially target TAMs while minimizing uptake by normal macrophages^27,28^.

In vitro*-*transcribed (IVT) mRNA has recently come into focus as a potential new drug class for delivering genetic information directly into existing cells^29^. These synthetic medicines can be engineered to induce the transient expression of selected proteins because they structurally resemble natural mRNA. They are easily developed, inexpensive to produce, and efficiently scalable for manufacturing purposes^30^. Advances in addressing the inherent challenges of this drug class, particularly related to controlling translational efficacy and immunogenicity of the IVT mRNA, have provided the basis for a broad range of potential applications^31–33^. In fact, clinical development of mRNA-based therapeutics has led to the formation of several university spin-off companies (for example, Argos Therapeutics, BioNTech, CureVac, eTheRNA, Ethris, Factor Bioscience, Moderna, and Onkaido), which are supported by considerable venture capital inflows to develop mRNA-based cancer immunotherapies and infectious disease vaccines^34^.

**Fig. 1:**
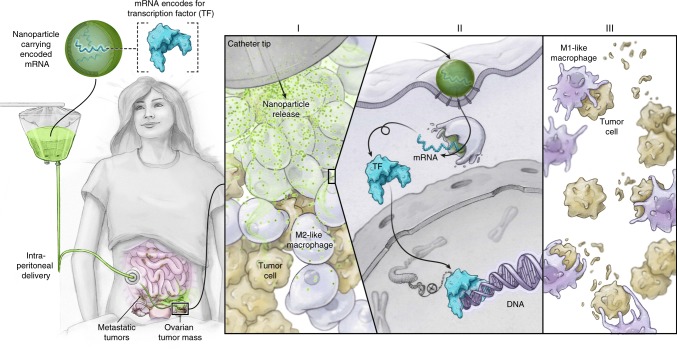
Transforming tumor-associated macrophages into tumoricidal cells using mRNA nanoparticles. We developed an injectable nanocarrier delivering in vitro-transcribed mRNA encoding M1-polarizing transcription factors as a method to rationally reprogram tumor-associated macrophages (TAMs) for therapeutic purposes without causing systemic toxicity. Illustrated is the first planned clinical application, designed to treat ovarian cancer patients with repeated intraperitoneal infusions of mRNA nanoparticles

Here, we explore the use of IVT mRNA formulated into an injectable therapeutic that can genetically re-program TAMs into antitumor macrophages without disrupting immune homeostasis or causing systemic toxicity (Fig. 1). We find that we can condense and protect IVT mRNA, and target the genes it carries to M2-like macrophages by formulating the message into biodegradable polymeric nanoparticles (NPs)^35^. To provide the targets with genes encoding master regulators of macrophage polarization, we first establish that co-expression of Interferon Regulatory Factor 5 (IRF5)^36^ and IKKβ (a kinase that phosphorylates and activates IRF5^37^) imprints TAMs with a potent pro-inflammatory and cytotoxic M1 phenotype. Using in vivo test systems that faithfully model advanced-stage ovarian cancer, metastatic melanoma, and glioblastoma, we establish that serial administration of IRF5/IKKβ-encoding NPs (via an intraperitoneal route for ovarian cancer, and injected intravenously to treat melanoma lung metastases or glioblastoma) substantially reduce tumor progression and, in some animals, even clear the disease. Phenotypic, functional, and gene expression studies reveal a dramatically reduced density of M2-like macrophages in tumor lesions of IRF5/IKKβ NP-treated mice compared to controls, along with increased numbers of inflammatory myeloid cells with distinct M1-type transcriptional profiles. Based on these data, our next step is to translate this technology into a clinical trial as an approach to treat ovarian cancer patients who were not responsive to other therapies. Although we will test IRF5/IKKβ NPs as a monotherapy first, our platform could ultimately reveal its full potential when used in synergy with existing immunotherapies, (e.g., T cell therapies, cancer vaccines, or checkpoint blockade inhibitors) by creating a therapeutic window for patients, thus stimulating a stronger overall immune response.

## Results

### Designing NPs to choreograph IVT mRNA transfection of TAMs

**Fig. 2:**
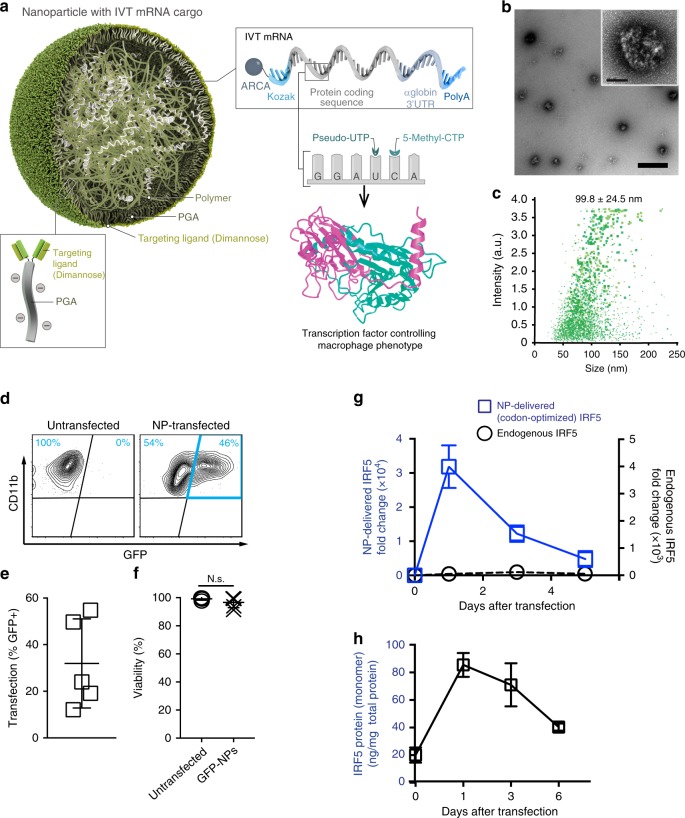
Mannose receptor-targeted mRNA nanoparticles efficiently transfect M2 macrophages. **a** Design of macrophage-targeted polymeric NPs formulated with mRNAs encoding key regulators of macrophage polarization. The particles consist of a PbAE-mRNA polyplex core coated with a layer of PGA-Di-mannose, which targets the particles to mannose receptors (CD206) expressed by M2-like macrophages. Also depicted is the synthetic mRNA encapsulated in the NP, which is engineered to encode the reprogramming transcription factors. **b** Transmission electron microscopy of a population of NPs (scale bar 200 nm) and a single NP (inset, scale bar 50 nm). **c** Size distributions, measured using a NanoSight NS300 instrument. **d**, **e** Gene-transfer efficiencies into bone marrow-derived macrophages measured by flow cytometry 24 h after nanoparticle transfection. *N* = 5 biologically independent samples. **f** Relative viability of NP-transfected and untransfected macrophages (assessed by staining with Annexin V and PI). N.S., non-significant. *N* = 5 biologically independent samples. **g** Expression kinetics of codon-optimized IRF5 mRNA (blue, left *Y* axis) and endogenous IRF5 mRNA (black, right *Y* axis) measured by qRT-PCR, *n* = 3 biologically independent samples for each time point. Shown are mean values ± SD. **h** Serial quantitative ELISA measurements of IRF5 protein (mean values ± SD, *n* = 3)

We developed a targeted mRNA delivery system that can introduce robust gene expression in the targeted cells by taking advantage of electrostatic interactions between cationic poly(β-amino ester) (PbAE) polymers and anionic mRNA (Fig. 2a). To improve the stability and translation of the mRNA encapsulated in the resulting nanocarriers, we used synthetic versions of the message that incorporate the modified ribonucleotides pseudouridine (Ψ)^32^ and 5-methylcytidine (m5C), and that are capped with ARCA (Anti-Reverse Cap Analog)^38^. Quality and purity of the mRNA was confirmed before use (Supplementary Fig. 1). The mRNA is released from the mRNA-PbAE complex intracellularly by hydrolytic cleavage of ester bonds in the PbAE backbone. We previously demonstrated efficient in vivo T cell transfection using this system^39^. To target the nanoparticles to TAMs as well as further stabilize the mRNA-PbAE complexes they contain, we engineered Di-mannose moieties onto their surfaces using polyglutamic acid (PGA) as a linker (Fig. 2a, Supplementary Fig. 2a). The NPs were manufactured utilizing a simple two-step, charge-driven self-assembly process. First, the synthetic mRNA was complexed with a positively-charged PBAE polymer, which condenses the mRNA into nano-sized complexes. This step was followed by the addition of PGA functionalized with Di-mannose, which shields the positive charge of the PBAE-mRNA particles and confers macrophage-targeting. The resulting mRNA nanocarriers had a size of 99.8 ± SE/24.5 nm (based on 3 independently manufactured batches), a polydispersity of 0.183, and an almost neutral surface charge (3.40 ± SE/2.15 mV ζ-potential, Fig. 2b, c). We first tested the transfection efficiency of our system for murine bone marrow-derived macrophages (BMDMs) using NPs formulated with green fluorescent protein-encoding mRNA (GFP-NPs). Briefly, 50,000 BMDMs were exposed to NPs containing 1 µg mRNA for 1 h, followed by flow cytometry measurements of GFP expression the next day. Following a single NP application, we routinely transfected 31.9% (±SE/8.5%; *n* = 7) of these primary macrophages without reducing their viability (Fig. 2d–f). Surface modification of particles with Di-mannose was relevant, as transfection rates with untargeted (but PGA-coated) nanocarriers dropped to an average of 25% (±SE/2.1%; *n* = 7) in this inherently phagocytic cell type.

### Programming immunosuppressive macrophages into proinflammatory phenotypes

To induce macrophage polarization, we next selected two mRNAs for inclusion into the NPs: the first encodes IRF5, a key member of the interferon regulatory factor family that favors the polarization of macrophages toward the M1 phenotype^36^; the second encodes IKKβ, a kinase that phosphorylates and activates IRF5^37^. We used a ratio of 3 IRF5 mRNAs to 1 IKKβ mRNA. Using real-time quantitative PCR specific for the NP-delivered (and codon-optimized) IRF5 mRNA, we found that mRNA expression in macrophages was maximal at day 1, resulting in a 1,500-fold increase in IRF5 relative to endogenous factor levels (Fig. 2g). As expected, gene expression was transient but IRF5 levels remained strongly upregulated through day 3 (581-fold increased) and day 5 (87-fold increased) before returning to baseline. IRF5 protein levels measured by ELISA showed a similar kinetic profile (Fig. 2h).

**Fig. 3:**
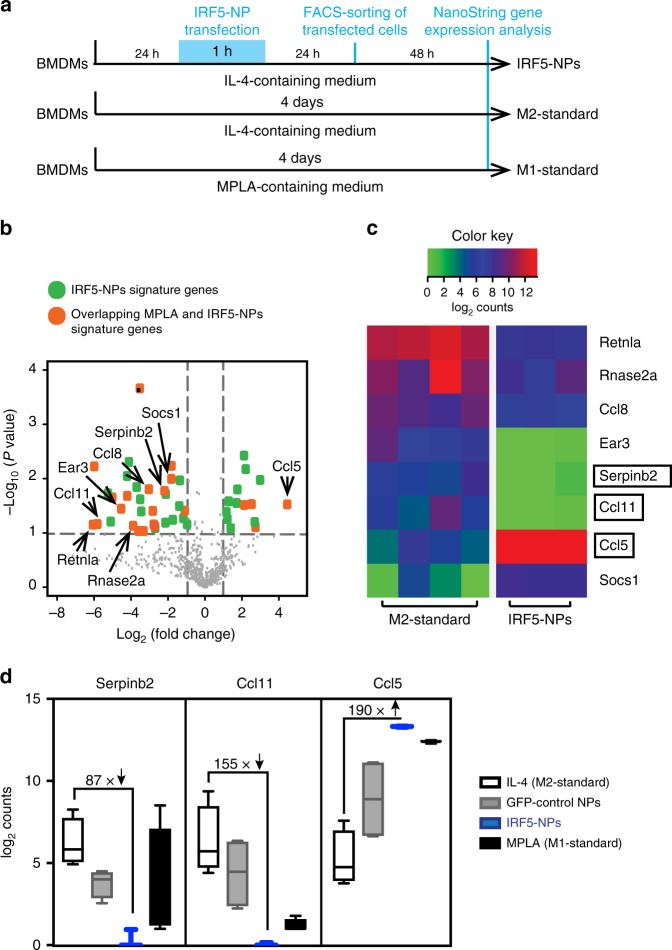
IRF5/ IKKβ mRNA nanoparticles can imprint a pro-inflammatory M1-like phenotype. **a** Timelines depicting NP transfection protocols and culture conditions for the BMDMs used in panels (**b**–**d**). **b** Gene expression profiles of IRF5/IKKβ NP-transfected macrophages compared to signature M1 cells stimulated with the Toll-like Receptor 6 agonist MPLA. Results are depicted as a Volcano plot that shows the distribution of the fold changes in gene expression. M1 signature genes are indicated. The *P* value of overlap between IRF5/IKKβ NP-transfected macrophages and the M1 signature gene set was determined by GSEA. **c** Heat map of M1 signature gene expression in macrophages cultured in IL-4 versus cells cultured in IL-4 and transfected with IRF5/IKKβ NPs. **d** Box plots showing counts for indicated genes. The boxes represent the mean values and the line in the box represents median. The bars across the boxes show the minimum and maximum values. Whiskers represent 95% confidence intervals. *N* = 5 biologically independent samples

To determine if IRF5/IKKβ-encoding NPs can reprogram M2 macrophages into the therapeutically desirable anti-cancer M1 phenotype, we used NanoString gene expression analysis. BMDMs were first cultured in the presence of interleukin-4 (IL-4) to induce a suppressive M2 phenotype (Fig. 3a). Following transfection with either control GFP-mRNA nanoparticles or IRF5/IKKβ mRNA-containing NPs, gene expression profiles were analyzed and compared with inflammatory macrophages, which we generated separately by exposing BMDMs to the TLR4 agonist Monophosphoryl Lipid A. We found that, despite being cultured in suppressive IL-4-containing medium, macrophages transfected with IRF5/IKKβ mRNA NPs display gene expression profiles similar to inflammatory macrophages (Fig. 3b). Signature M2 macrophage genes, such as Serpinb2 and Ccl11, were strongly downregulated while key M1 differentiation genes, such as Ccl5, were upregulated (Fig. 3c, d). These data establish that NP-mediated expression of IRF5 and its kinase skews suppressive macrophages toward a proinflammatory phenotype.

### Therapeutic effects of NP-delivered pro-M1 genes for disseminated ovarian cancer

**Fig. 4:**
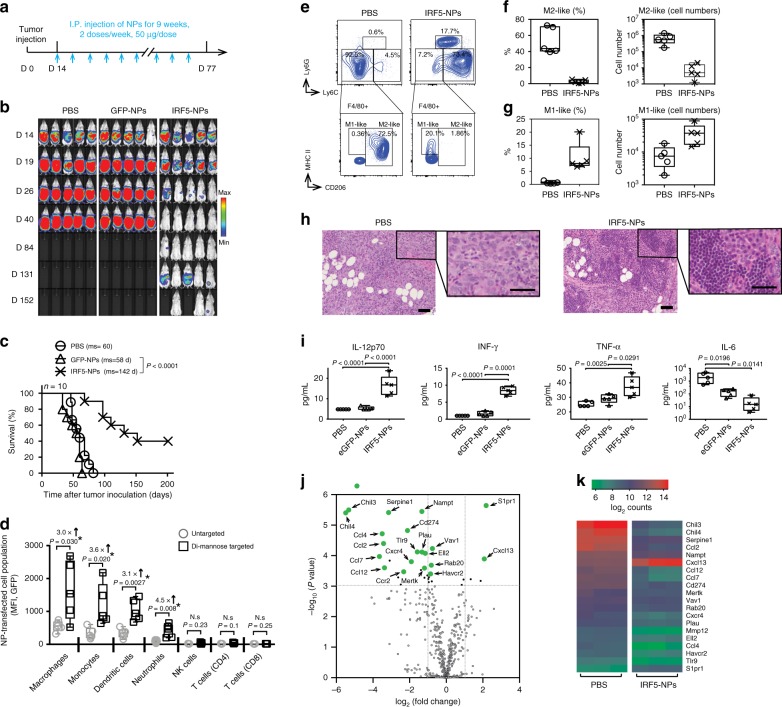
IRF5/IKKβ mRNA nanoparticles double survival of mice with ovarian cancer. **a** Time lines and dosing regimens. **b** Sequential bioluminescence imaging of tumor growth. **c** Kaplan–Meier survival curves. Statistical analysis was performed using the log-rank test. *N* = 10 biologically independent animals. **d** Flow cytometric quantitation of in vivo transfection rates in different immune cell subpopulations 48 h after a single i.p. dose of untargeted versus Di-mannose-coated NPs carrying GFP mRNA: macrophages (CD45+, CD11b+, MHCII+, CD11c-, Ly6C-/low, Ly6G-), monocytes (CD45+, CD11b+, MHCII+, CD11c-, Ly6C+, Ly6G-), dendritic cells (CD45+, CD11c+, CD11b-, MHCII+), neutrophils (CD45+, CD11b+, MHCII+, CD11c-, Ly6G+), CD4+ T cells (CD45+, TCR-β chain+, CD4+, CD8-), CD8+ T cells (CD45+, TCR-β chain+, CD4-, CD8+), and natural killer cells (CD45+, TCR-β chain-, CD49b+) were measured. **e** Flow cytometric analysis of macrophage phenotypes in the peritoneum of mice with disseminated ID8 ovarian cancer. Animals were either treated with 4 doses of IRF5/IKKβ NPs or PBS. **f** Box plots summarizing relative percent (left panel) and absolute numbers (right panel) of Ly6C-, F4/80+, and CD206+ (M2-like) macrophages. Corresponding numbers for Ly6C-, F4/80+, and CD206- (M1-like) macrophages are shown in (**g**). **h** Representative hematoxylin and eosin (H&E)-stained sections of ovarian tumor-infiltrated mesenteries isolated from PBS controls (left panel) or IRF5/IKKβ NP-treated animals (right panel; scale bar 100 µm). Tenfold magnifications of representative malignant lesions are shown on the right (scale bar 50 µm). **i** Luminex assay measuring cytokines produced by isolated peritoneal macrophages from each treatment group. In parallel experiments, FACS-sorted CD11b+, F4/80+ peritoneal macrophages were directly analyzed by NanoString gene expression analysis. **j** Results are depicted as a Volcano plot. **k** Heat map of signature gene expression in macrophages isolated from mice treated with IRF5-NPs versus control PBS. All boxes in boxplots in this figure represent the mean values and the line in the box represents median. The bars across the boxes show the minimum and maximum values. Whiskers represent 95% confidence intervals. *N* = 5 biologically independent samples

To evaluate this treatment approach in a clinically-relevant in vivo test system, we used a model that recapitulates late-stage, unresectable ovarian tumors in C57BL/6 mice; these animals are injected with ID8 ovarian cancer cells which were tagged with luciferase to enable serial bioluminescent imaging of tumor growth. The tumors were allowed to establish for two weeks. By this stage, the mice have developed nodules throughout the peritoneal wall and in the intestinal mesentery. The animals were divided into three groups that received PBS (control), GFP-NPs (sham), or IRF5/IKKβ NP treatment at an i.p. dose of 100 μg mRNA/mouse/week for 9 weeks (Fig. 4a). We observed that in the IRF5/IKKβ NP treated group, the disease regressed and was eventually cleared in 40% of animals (overall 142 d median survival versus 60 d in controls; Fig. 4b, c). To understand the underlying mechanisms of IRF5/ IKKβ NP-mediated anti-tumor effects, we first examined how exclusively mannose receptor-targeting confined NP interaction to phagocytes. Flow cytometry of peritoneal lavage fluid collected 24 h after one dose of NPs established that Di-mannose coated nanocarriers preferentially transfect macrophages and monocytes, but also at low levels dendritic cells and neutrophils (all cell types with described CD206 expression, (Fig. 4d)). In contrast, gene transfer into CD206-negative cells, such as NK cells or T cells, was undetectable. We next conducted a detailed phenotypic and functional analysis of macrophage/monocyte populations in the peritoneum of mice with established ovarian cancer following treatment with IRF5/IKKβ nanoparticles or PBS over a 3-week period (two weekly injections). Flow cytometric analysis revealed that IRF5/IKKβ NPs reduced the population of immune-suppressive macrophages (Ly6C-, F4/80+, CD206+) to an average 2.6% ± SE/2.1% (*n* = 5) versus 43% ± SE/15.6% in controls (Fig. 4e, f; Supplementary Fig. 3). Conversely, the fraction of M1-like macrophages increased from 0.5% ± SE/0.2% to 10.2% ± SE/4.1% (Fig. 4e, g). IRF5 gene therapy also affected the population of other immune cells. In particular, inflammatory monocytes (CD11b+, Ly6C+, Ly6G−) were more abundant (73.4% ± SE/3.6% compared to 4.5% ± SE/1.9% in untreated mice). One interesting finding in all IRF5 NP-treated animals was multifocal dense clusters of lymphocytes present within or surrounding the neoplasms (Fig. 4h), indicating that genetic programming of immune-stimulatory macrophages may restore lymphocyte migration and infiltration into solid tumors.

We isolated peritoneal macrophages by fluorescence-activated cell sorting to analyze their cytokine secretion, and detected a robust increase in the release of pro-inflammatory (anti-tumor) cytokines IL-12 (3.4-fold higher), IFN-γ (8.4-fold higher), and TNF-α (1.5-fold higher), whereas the levels of IL-6, a regulatory cytokine associated with differentiation toward alternatively activated (M2-like) macrophages, were reduced by 97-fold; Fig. 4i). Genome expression profiling confirmed differentiation toward an M1-like macrophage phenotype in IRF5/IKKβ nanoparticle-treated mice (Fig. 4j, k; Supplementary Fig. 4).

### Role of host T cells

**Fig. 5:**
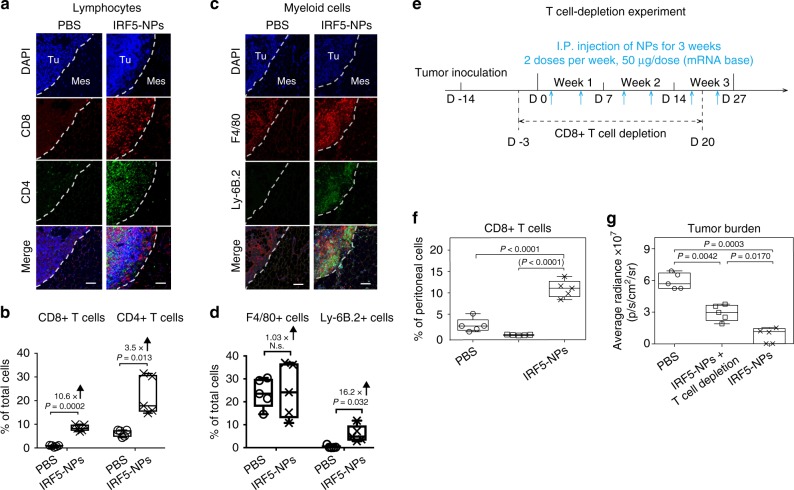
T cells contribute to anti-tumor effects achieved with macrophage-programming nanoparticles. **a**–**e** Nanoparticle-mediated macrophage programming increases T cell recruitment into tumor lesions. Shown are representative confocal images of peritoneal metastases of ID8 ovarian cancer cells in the mesentery. Tissues were collected after 6 biweekly i.p. injections of PBS or IRF5/IKKβ NPs (50 μg mRNA/dose), and were stained for the indicated lymphocyte- and myeloid-markers (**a**, **c**). Tu = Tumor, Mes = Mesentery. Scale bar: 100 μm. **b**, **d** Box plots showing fluorescent signals for each phenotypic marker using Halo™ image analysis software. *N* = 5. The boxes represent the mean values and the line in the box represents median. The bars across the boxes show the minimum and maximum values. Whiskers represent 95% confidence intervals. *N* = 5 biologically independent samples. **e** Experimental design for CD8+ T cell depletion studies in C57BL/6 mice with disseminated ID8 ovarian cancer. To deplete CD8+ T cells, mice were injected i.p. with 1 mg anti-CD8b mAb (YTS169.4) three days before the first nanoparticle administration, followed by one 0.5 mg dose every five days for a total of six doses. **f** Flow cytometry of peritoneal cells collected on day 27 established efficient depletion of CD8+ T cells (average 98.7%). **g** Plots of ID8 tumor luciferase signal intensities after six nanoparticle injections (day 27). On each box plot, the central mark indicates the median, and the bottom and top edges of the box indicate the interquartile range. Whiskers represent 95% confidence intervals. *N* = 5 biologically independent samples. Pairwise differences in photon counts between treatment groups were analyzed using the Wilcoxon rank-sum test. Shown are data for five mice per treatment condition pooled from two independent experiments

To study in more detail how NP-mediated macrophage reprogramming affects the tumor immune cell composition, we phenotyped lymphocytes and myeloid-cells in mesenteric ovarian cancer lesions by confocal microscopy. We found that IRF5/IKKβ NPs increased T cell infiltration into tumors by an average 10.6-fold (CD8) and 3.5-fold (CD4; Fig. 5a, b). Also, densities of neutrophils increased by 16.2-fold (Fig. 5c, d). To measure how much the anti-tumor responses we achieved with IRF5/IKKβ NP macrophage reprogramming were mediated by host T cells, we next used monoclonal antibodies to deplete CD8+ T cells in mice with established ID8 ovarian cancer (Fig. 5e, f). Therapeutic responses between fully immunocompetent and lymphocyte-depleted mice were compared after six IRF5/IKKβ NP doses (D27) using bioluminescence tumor imaging. In the absence of T cells, macrophage-programming IRF5/IKKβ NPs still induced 28.4% (±SE/3.5%; *n* = 5) of the anti-tumor activity observed in the presence of T cells (Fig. 5g). This indicates that T cells contribute to, but are not the sole mediators of, the anti-tumor effects achieved with macrophage-programming nanoparticles.

### Biodistribution and safety

**Fig. 6:**
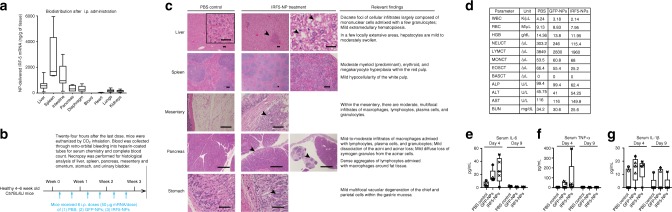
Macrophage-programming mRNA nanocarriers are biocompatible and safe for repeated i.p. dosing. **a** In vivo biodistribution of macrophage-targeted IRF5/IKKβ NPs following i.p. administration. NP-delivered (codon optimized) mRNA was detected by qPCR 24 h after a single injection of particles containing 50 μg mRNA. The boxes represent the mean values and the line in the box represents median. The bars across the boxes show the minimum and maximum values. Whiskers represent 95% confidence intervals. *N* = 19 biologically independent animals. **b**–**d** These panels summarize a pathology report prepared by a Comparative Pathologist at *FHCRC*. **b** Schematic representation of the experimental timeline. **c** Representative H&E-stained sections of various organs isolated from controls or NP-treated animals. Scale bar, 100 µm. Lesions found in the NP-treated animals are shown and described on the right. **d** Serum chemistry and blood counts. Luminex assay measurements of serum IL-6, TNF-α, and IL-1β cytokines 4 or 9 days after a single i.p. injection of IRF5/IKKβ NPs are shown panels (**e**), (**f**), and (**g**), respectively. On each box plot, the central mark indicates the median, and the bottom and top edges of the box indicate the interquartile range. Whiskers represent 95% confidence intervals. *N* = 5 biologically independent samples

We next quantified the distribution of nanoparticles in various organs 24 h after intraperitoneal injection using RT-qPCR assays designed to detect only nanoparticle-delivered (codon optimized) IRF5. The highest concentrations of IVT mRNA were found in organs located in the peritoneum, including liver, spleen, intestine, pancreas, and diaphragm (Fig. 6a). We also detected small amounts of particle-delivered mRNA in organs that lie outside of the peritoneum (heart, lungs, kidneys), suggesting that a fraction of i.p. injected nanocarriers entered the systemic circulation. Guided by the distribution data, we next assessed whether these nanoreagents are biocompatible and safe for repeated dosing. Mice were injected with a total of eight doses of IRF5/IKKβ NPs (two 50 μg mRNA doses/week for 4 weeks, Fig. 6b). They were euthanized 24 h after the final dose, body weight was recorded, blood was collected by retro-orbital bleed for serum chemistry, and a complete gross necropsy was performed. There was no difference in body weights or body temperatures between groups (Supplementary Fig. 5). The following tissues were evaluated by a board-certified staff pathologist: liver, spleen, mesentery, pancreas, stomach, kidney, heart, and lungs. Histopathological evaluation revealed in all cases multifocal dense clusters of lymphocytes within or surrounding tumor lesions, but no evidence of inflammation or frank necrosis was observed in tissues where neoplastic cells were not present (Fig. 6c). Also, serum chemistry of IRF5/IKKβ NP-treated mice was comparable to that of PBS controls, indicating that systemic toxicities did not occur (Fig. 6d). Because we detected small amounts of IRF5-mRNA systemically in our biodistribution studies, we designed parallel experiments to quantitate inflammatory cytokines in the peripheral blood. Following a single i.p. injection of IRF5/IKKβ NPs, we measured a moderate and transient increase in serum levels of interleukin-6 (IL-6) to an average of 26.8 pg/mL (Fig. 6e), tumor necrosis factor-α (TNF-α) to an average of 94.7 pg/mL (Fig. 6f), and interleukin-1beta (IL-1β) to an average of 14.1 pg/mL (Fig. 6g). Based on previous reports, these levels are around 500-fold lower than those associated with pathological findings and thus can be considered safe^40,41^.

### Controlling systemic tumor metastases with intravenous infusions of IRF5/IKKβ nanoparticles

**Fig. 7:**
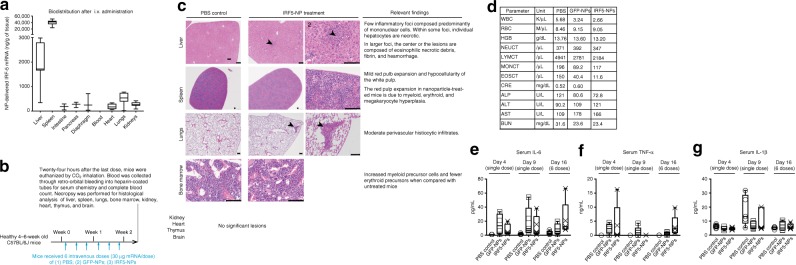
Repeated intravenous infusions of nanocarriers are not associated with systemic toxicities. **a** In vivo biodistribution of macrophage-targeted IRF5/IKKβ NPs following i.v. administration. Codon-optimized mRNA was measured by qPCR 24 h after a single i.v. injection of particles containing 50 μg mRNA. The boxes represent the mean values and the line in the box represents median. The bars across the boxes show the minimum and maximum values. Whiskers represent 95% confidence intervals. *N* = 19 biologically independent animals. **b**–**d** These panels summarize a pathology report prepared by a Comparative Pathologist at *FHCRC*. **b** Schematic representation of the experimental timeline. **c** Representative H&E-stained sections of various organs isolated from controls or NP-treated animals. Scale bars, 100 µm. Lesions found in the NP-treated animals are shown and described on the right. **d** Serum chemistry and blood counts. Luminex assay measurements of serum IL-6, TNF-α, and IL-1β cytokines 4 or 9 days after a single i.v. injection and 2 days after 6 repeated doses (Day 16) of IRF5/IKKβ NPs are shown panels (**e**), (**f**), and (**g**) respectively. On each box plot, the central mark indicates the median, and the bottom and top edges of the box indicate the interquartile range. Whiskers represent 95% confidence intervals. *N* = 5 biologically independent samples

Based on the therapeutic responses we achieved with IRF5/IKKβ NPs administered directly into the peritoneal cavity to treat tumor lesions spread throughout the peritoneum, we next asked whether intravenously infused mRNA nanocarriers could program macrophages systemically to control disseminated disease. RT-qPCR biodistribution studies revealed that i.v.-infused nanocarriers preferentially deliver their mRNA cargo to organs with high levels of resident macrophages/phagocytes, mostly the spleen, liver, and lungs (Fig. 7a). A full histopathological examination of mice after six doses of intravenously infused nanoparticles (30 µg/dose, Fig. 7b) revealed only moderate mononuclear infiltrates in the lungs and liver as well as mild red pulp expansion of the spleen due to myeloid, erythroid, and megakaryocyte hyperplasia (Fig. 7c). Importantly, overall liver function was normal in all IRF5/IKKβ NP-treated mice, with only minimally elevated blood levels of the liver enzymes alanine transaminase (ALT) and aspartate aminotransferase (AST, Fig. 7d). Serum creatinine (CRE) levels were unaltered by nanoparticle infusions, indicating normal renal function (Fig. 7d). In addition, nanoparticle treatments caused only modest increases in the expression levels of inflammatory cytokines (Fig. 7e–g). Taken together, these results indicate IRF5/IKKβ NPs are biocompatible and safe for repeated intravenous dosing. The most prominent histological lesions (mononuclear infiltrates within the parenchyma of the lung and liver) were minor reactions that typically resolve with minimal or no clinical intervention^42^.

**Fig. 8:**
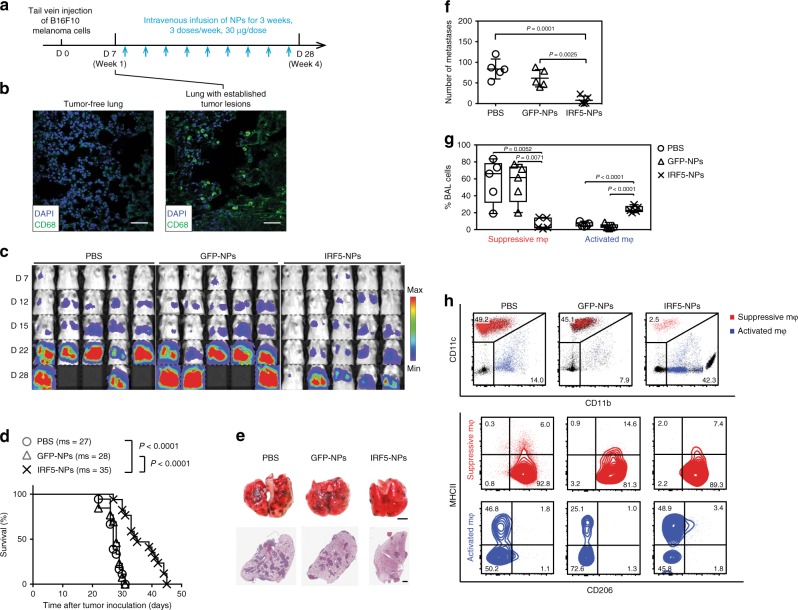
Intravenously infused IRF5/IKKβ nanoparticles can control tumor metastases in the lung. **a**–**g** C57BL/6 albino mice were injected via the tail vein with 1 × 10^6^ B16F10 firefly luciferase-expressing melanoma cells to establish lung metastases. After 7 days, animals were randomly assigned to either the IRF5/IKKβ NP treatment group, a control GFP NP group, or a PBS control. **a** Time lines and dosing regimens. **b** Confocal microscopy of healthy lungs (left panel) and B16F10 tumor-infiltrated lungs (right panel). Infiltrating macrophage populations fluoresce in green. **c** Sequential bioluminescence tumor imaging. **d** Kaplan–Meier survival curves for each treatment group. ms, median survival. Statistical analysis was performed using the log-rank test, and *P* < 0.05 was considered significant. *N* = 10 biologically independent animals. **e** Representative photographs (top row) and micrographs of lungs containing B16F10 melanoma metastases representing each group following 2 weeks of treatment. Counts of lung tumor foci are summarized in (**f**). *N* = 5 biologically independent samples. **g** Phenotypic characterization of monocyte/macrophage populations in bronchoalveolar lavage from each treatment group (*n* = 5 biologically independent samples). The relative percentages of suppressive and activated macrophages are summarized by the data shown in (**h**)

To measure anti-tumor responses in a clinically relevant in vivo test system, we administered particles containing IRF5/ IKKβ mRNA into mice with disseminated pulmonary melanoma metastases (Fig. 8a). Recent work describes the foundational role of monocytes and macrophages in establishing metastases caused by this disease^43,44^, and we confirmed by confocal microscopy that tumor engraftment was coordinate with phagocyte accumulation in the lungs (Fig. 8b). Tumor burdens were determined by bioluminescent imaging, and mice with detectable cancers were sorted into groups that had matching levels. Groups were then randomly assigned to treatment conditions, receiving no therapy (PBS), or intravenous injections of GFP- or IRF5/ IKKβ-encapsulating nanoparticles. We found that only IRF/IKKβ nanoparticle therapy substantially reduced tumor burdens in the lungs; in fact, they improved overall survival by a mean 1.3-fold (Fig. 8c, d). In parallel experiments, mice were sacrificed 22 days after tumor inoculation to validate bioluminescence tumor signals with counts of pulmonary metastases and to assess macrophage polarization by flow cytometry. The total number of metastases in the lungs of IRF5/IKK NP-treated animals was 8.7-fold reduced (average 40 ± SE/16 metastases; *n* = 5) compared to PBS controls (average 419 ± SE/139 metastases; Fig. 8e, f). Flow cytometry of bronchoalveolar lavage fluid cells revealed a strong shift from immune-suppressive (CD206+, MHCII−, CD11c+, CD11b^low^) macrophages (displayed in red) toward activated (CD206-, MHCII+, CD11c−, CD11b+) phagocytes (shown in blue, Fig. 8g, h).

### Programming tumor-suppressing phagocytes to treat glioma

**Fig. 9:**
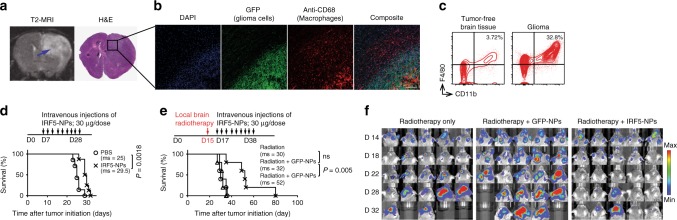
Macrophage reprogramming improves the outcome of radiotherapy in glioma. **a** T2 MRI scan, and histological staining following initiation of a PDGFβ-driven glioma in RCAS-PDGF-B/Nestin-Tv-a; *Ink4a/Arf*−/−; *Pten−/−* transgenic mice on post-induction day 21. **b** Confocal microscopy of CD68+ TAMs infiltrating the glioma margin. Scale bar 300 µm. **c** Flow cytometry analysis of macrophage (F4/80+, CD11b+) populations in healthy brain tissue versus glioma. **d**, **e** Kaplan–Meier survival curves of mice with established gliomas receiving IRF5/IKKβ treatments as a monotherapy (**d**) or combined with brain tumor radiotherapy (**e**). Time lines and dosing regimens are shown on top. Ms, median survival. Statistical analysis was performed using the log-rank test, and *P* < 0.05 was considered statistically significant. *N* = 5 biologically independent animals. **f** Sequential bioluminescence imaging of tumor progression

For a third in vivo test system we examined glioma, which is a difficult to manage cancer type where M2-like macrophages represent the majority of non-neoplastic cells and promote tumor growth and invasion^45^. Currently, the standard of care for this disease is radiotherapy, which unfortunately offers only a temporary stabilization or reduction of symptoms and extends median survival by just 3 months^46^. To recapitulate the genetic events and subsequent molecular evolution of the disease, we used the RCAS-PDGF-B/Nestin-Tv-a; *Ink4a/Arf*−/−; *Pten−/−* transgenic mouse model of PDGFβ-driven glioma (PDG mice^47,48^). Brain tissue was stereotactically injected with a mixture of DF-1 cells transfected with either RCAS-PDGFβ or RCAS-cre retrovirus (1:1 mixture, 2 µL). Overexpression of the PDGFβ oncogene and the absence of the tumor suppressor genes Ink4a-arf and Pten in glioma progenitors led to the formation of 4–5 mm diameter tumors (Fig. 9a) with a nearly complete penetrance within 21 days (as established previously^47^). Using immunofluorescence, we confirmed the presence of tumor-infiltrating (CD68^+^) macrophages (Fig. 9b, indicated in red) in established gliomas (shown in green). Flow cytometry revealed that the F4/80+, CD11b+ macrophage population accounted for 32.8% of total cells in the tumor, which is ninefold higher than seen in age-matched healthy control mice (3.7%) (Fig. 9c). The PDG mice in our experiments express the firefly luciferase gene linked to a key cancer gene promoter. Bioluminescence from this reporter has been demonstrated to be positively correlated with tumor grade^49^, so we used it to monitor tumor development every four days after the onset of treatment. We first tested IRF/IKKβ NPs as a monotherapy: PDG mice received intravenous infusions of 9 doses of NPs loaded with IRF5/IKKβ mRNA, or PBS in the control group (3 doses/week for 3 weeks). We first found that IRF/IKKβ NP treatments only modestly suppressed tumor progression (producing on average only a 5-day survival advantage compared to untreated controls; Fig. 9d). However, combining radiotherapy as the standard-of-care with IRF5/IKKβ NP injections substantially reduced tumor growth and more than doubled the survival of treated mice compared to the PBS control group (52 days versus 25 days, respectively; Fig. 9e, f).

In conclusion, our in vivo results from three preclinical solid tumor models demonstrate that nanoparticles, administered either locally or systemically, can deliver genes encoding master regulators of macrophage polarization to re-program immunosuppressive macrophages into tumor-clearing phenotypes.

### Translation from murine to human macrophages

**Fig. 10:**
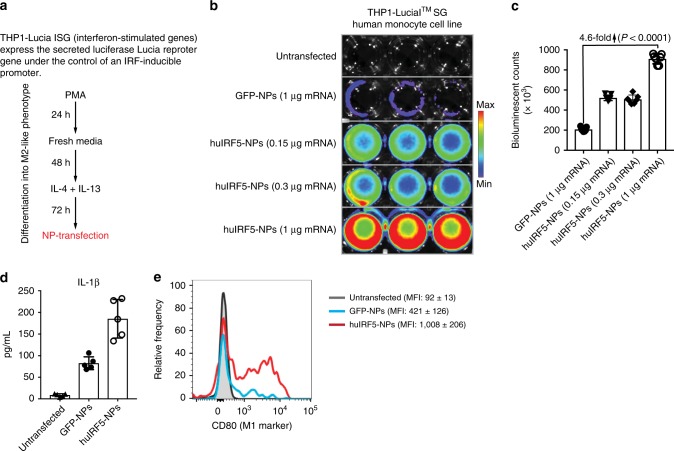
MRNA-carrying nanoparticles encoding human IRF5/IKKβ reprogram human macrophages. **a** Time line and culture conditions to differentiate the human THP-1 monocytic cell line into suppressive M2-like macrophages. **b** Bioluminescent imaging of M2-differentiated THP1-Lucia cells cultured in 24-well plates and transfected with indicated concentrations of NPs carrying human IRF5/ IKKβ mRNA versus control GFP mRNA. Shown are three representative wells per group. Levels of IRF-induced Lucia luciferase were determined 24 h after transfection using Quanti-Luc. Bioluminescent counts are summarized in the bar graph shown in (**c**). *N* = 5 biologically independent samples. Differences in IL-1β cytokine secretion and surface expression of the M1-macrophage marker CD80 are shown in (**d**) and (**e**), respectively. Shown are mean values ± SD

To confirm that our data acquired in mice have relevance to treat human disease, we fabricated NPs delivering IVT mRNA encoding human IRF5 and IKKβ (huIRF5 NPs). We used the human monocytic cell line THP-1 as a well-established M1 and M2 macrophage polarization model to test these nanocarriers^50,51^. M2-type macrophages were generated by treating THP-1 cells with PMA and polarizing them with IL-4 and IL-13 (Fig. 10a). To confirm that huIRF5 NPs are functional and activate the IRF pathway, we transfected THP1-Lucia^TM^ ISG cells with nanoparticles loaded with huIRF5/IKKβ or GFP control mRNAs. THP1-Lucia^TM^ ISG cells secrete the fluorescent Lucia reporter under the control of an IRF-inducible promoter. This composite promoter is comprised of five IFN-stimulated response elements (ISRE) fused to an ISG54 minimal promoter, which is unresponsive to activators of the NF-κB or AP-1 pathways. As a result, THP1-Lucia™ ISG cells allow the monitoring of the IRF pathway by determining the activity of the Lucia luciferase. We found that huIRF5 NPs strongly upregulated luciferase expression in M2-polarized THP-1 cells, indicating that the mRNA constructs we designed are functional in human cells (Fig. 10b, c). To determine whether IRF5 pathway activation can re-program M2-polarized THP-1 cells toward an M1-like phenotype, we measured secretion of the pro-inflammatory cytokine IL-1β following NP transfection. Production of IL-1β was significantly increased in THP-1 cells transfected with huIRF5 NPs versus untransfected controls (mean 21-fold; *P* < 0.0001; unpaired, two-tailed Student’s *t* test; Fig. 10d), which correlated with a robust upregulation (10.9-fold increased MFI, *P*<0.0001; unpaired, two-tailed Student’s *t* test) of the M1 macrophage cell surface marker CD80 (Fig. 10e). Based on these data, we began working with the *Nanotechnology Characterization Laboratory* at the *National Cancer Institute* to further characterize the quality and biocompatibility of our nanocarriers, according to FDA regulations for nanomedicine with the goal of submitting an IND for a first-in-human clinical trial to test with chemotherapy-resistant ovarian carcinoma patients.

## Discussion

In vitro transcribed (IVT) mRNA is gaining momentum as a potential new drug class for induction of antigen-specific immunity, especially in the field of immuno-oncology^52^. IVT has also been used as a starting material for cell therapy approaches aimed at gene editing or immune interventions^53–55^. Here, we explored the use of IVT mRNA to reprogram TAMs as a strategy to treat cancer. We demonstrate that suppressive M2 macrophages can be genetically reconfigured in situ into tumor-clearing M1 cells by nanoparticles targeted to provide them with genes encoding master regulators of macrophage polarization.

For our proof-of-concept, we chose to express IRF5 and its activating kinase IKKβ rather than other members of the IRF protein family that can induce M1 differentiation to subsets (e.g., IRF1 or IRF8) because of the diverse impact IRF5 has on the activation of genes encoding type I interferon, inflammatory cytokines, and tumor suppressors^36,56^. However, our group is also interested in testing whether combinations of IRFs (e.g., IRF5 and IRF8) can synergistically improve the anti-tumor potential we describe here. Furthermore, engineered versions of IRF proteins with supra-physiological function have been reported, and we will examine these before moving the technology into clinical trials^57,58^. Because some IRF proteins (e.g., IRF3 and IRF4) can induce inhibitory M2 phenotypes^59^, the approach we describe here could also be used to develop injectable nanomedicine configured to treat autoimmune diseases where inflammatory macrophages play key roles in the pathogenesis, such as systemic lupus erythematosus, rheumatoid arthritis, Sjogren's syndrome, multiple sclerosis, and inflammatory bowel disease^60^. Our platform is therefore highly adaptable and could be explored beyond cancer therapy for a wide range of disease types that are caused or facilitated by macrophages.

The structural and chemical modifications we integrated into the nanoparticle-delivered IVT mRNA constructs (depicted in Fig. 2a) have been used before to ensure intracellular stability, translational efficiency, and low immunogenicity. In particular, the incorporation of the modified nucleosides pseudouridine and 5-methylcytidine into IVT mRNA has been shown to substantially reduce immune stimulation and stabilize the molecule against RNase cleavage^32,61^, and these modifications have been shown to improve cytosolic persistence and protein expression^32^.

Immune responses generated by unmodified mRNA can be beneficial for vaccination purposes, which is currently one of the major clinical applications of mRNA drugs^52,62^. These self-adjuvanted vaccines are administered subcutaneously or intramuscularly, where they locally induce innate immune responses. However, for our technology, nonspecifically-induced innate immune effects comprise a detriment: in situ reprogramming of macrophages relies on systemic or intraperitoneal infusions of mRNA therapeutics, which means unmodified mRNA constructs would cause wide-spread inflammatory toxicities and thus translational shutdown and premature degradation of the RNA. We demonstrate in Figs. 6 and 7 that IRF5/IKKβ NPs incorporating modified mRNAs are safe for repeated dosing, with only modest increases in the expression levels of inflammatory cytokines. To further improve clinical safety, the pseudouridine nucleotides used in our studies could be replaced with the recently developed N1-methyl-pseudouridine (N1mψ) analog, which further reduces immunogenicity and at the same time improves translation of mRNA therapeutics^63^.

Our choice for the nanocarrier substrate was primarily guided by safety, along with compatibility with scale-up production under GMP-conditions to facilitate carrying this nanomedicine forward into human studies. Our group tested a panel of cationic materials, including hyperbranched STAR Polymer^64^, polyethylene glycol-grafted polyethylenimine^65^, and mesoporous silica nanoparticles^66^, and selected PBAE 447 based on its superior transfection efficacy and low biomaterial-mediated cytotoxicity in primary macrophages. The safety of PBAE 447 is related to its high biodegradability, resulting in a half-life between 1 and 7 h in aqueous conditions^67^. This time frame is ideal for mRNA gene therapy, as the polymer condenses and effectively protects nucleic acids against degradation while they are within the endosome, but releases them once the nanoparticles come in contact with the cytoplasm. These nanocarriers can be manufactured using a fast (<10 min) two-step, charge-driven self-assembly process. In the first step, IVT mRNA is complexed with a positively-charged PBAE polymer, which condenses the mRNA into nano-sized complexes (Fig. 2b, c). This is followed by the addition of pre-formed PGA functionalized with Di-mannose, which shields the positive charge of the PBAE-mRNA particles and confers M2 macrophage-targeting. Using a fully-automated continuous-flow microfluidics system, we can now manufacture these nanodrugs with nearly uniform physical and functional characteristics at clinical scales.

At this point, it isn’t difficult to predict the ways our system can extend beyond a carrier that conveys IVT mRNA across the cell membrane of macrophages. For example, we know from our therapeutic studies that administration of control nanoparticles delivering mRNA encoding GFP instead of IRF5/IKKβ is insufficient to induce significant therapeutic benefits (Fig. 4b, Fig. 8d, e, Fig. 9f). In fact, gene expression analysis of GPF NP-transfected M2-like macrophages revealed a slight shift toward the M1 phenotype (Fig. 3b–d), suggesting that the mere uptake of these neutrally charged particles may induce signals that facilitate M1 polarization.

As expected for a therapeutic approach that does not directly target or lyse tumor cells, IRF5/IKKβ programming of macrophages delayed tumor progression but did not eradicate the disease in the majority of treated animals. Considering the substantial role TAMs play in cancer proliferation, angiogenesis, invasion, and metastasis^44^, we suspect that our platform could be used to its best advantage as a companion therapeutic for patients that are refractory to other treatments such as immune checkpoint inhibitors, cancer vaccines, T cell therapies, or antibody approaches.

Our first clinical translation of this technology at *Fred Hutchinson Cancer Research Center* will be as a monotherapy administered directly via intraperitoneal catheter to treat ovarian cancer patients (as illustrated in Fig. 1). Intraperitoneal chemotherapy, given in cycles over 6–9 weeks, is now standard of care for ovarian cancer patients^68,69^, which means clinical protocols are already in effect on how to best introduce, maintain, and eventually remove the catheter. The advantages to delivering nanoparticles directly into the abdominal cavity in the first Phase 1 trial are: (i) increased safety (limited systemic exposure, and the option to drain and flush the peritoneal cavity as required), (ii) high volume (up to 1 L of nanoparticles can be safely administered via catheter within minutes), and (iii) direct physical targeting (as ovarian cancer lesions are usually confined within the peritoneal cavity). The results of these investigations will provide important information for the design of Phase 1 trials for systemic applications aimed at treating less-focalized forms of cancer.

## Methods

### Cell lines

The murine ovarian cancer cell line ID8, a gift from Dr. Katherine Roby (University of Kansas Medical Center, Kansas City, KS)^70^, was cultured in DMEM supplemented with 4% FBS and 5 μg/ml insulin, 5 μg/ml transferrin, and 5 ng/ml sodium selenite (all Sigma-Aldrich). To generate the more aggressive vascular endothelial growth factor (VEGF)-expressing strain, we transfected ID8 tumor cells with the pUNO1 plasmid (Invivogen) encoding murine VEGF and the blasticidin-resistance gene. To obtain stable transfectants, tumor cells were cultured in complete medium containing 10 μg/ml Blasticidin (InvivoGen) for three weeks. The THP1-Lucia^TM^ ISG cells (interferon regulatory factor-inducible reporter monocytes) were purchased from InvivoGen (Cat# thp1-isg) and cultured in RPM1–1640 supplemented with 0.05 mM 2-mercaptoethanol and 10% fetal bovine serum (FBS). All cell lines tested negative for mycoplasma based on DNA-based PCR tests (DDC Medical).

### PbAE synthesis

We combined 1,4-butanediol diacrylate with 4-amino-1-butanol in a 1:1 molar ratio of diacrylate to amine monomers. Acrylate-terminated poly(4-amino-1-butanol-co-1,4-butanediol diacrylate) was formed by heating the mixture to 90 °C with stirring for 24 h. 2.3 g of this polymer was dissolved in 2 mL tetrahydrofuran (THF). To form the piperazine-capped 447 polymer, 786 mg of 1-(3-aminopropyl)−4-methylpiperazine in 13 mL THF was added to the polymer/THF solution and stirred at room temperature (RT) for 2 h. The capped polymer was precipitated with 5 volumes of diethyl ether, washed with 2 volumes of fresh ether, and dried under vacuum for 1 day. Neat polymer was dissolved in dimethyl sulfoxide (DMSO) to a concentration of 100 mg/mL and stored at −20 °C.

### PGA conjugation to Di-mannose

α-D-mannopyranosyl-(1→2)-α-D-mannopyranose (Di-mannose, Omicron Biochemicals Inc.) was modified into glycosylamine before being conjugated to PGA. First, the Di-mannose (157 mg) was dissolved in 10.5 mL of saturated aqueous ammonium carbonate, then stirred at RT for 24 h. On the second day, more solid ammonium carbonate was added until the Di-mannose precipitated from the reaction solution. The mixture was stirred until completion, as measured by TLC, followed by lyophilization to remove the excess ammonium carbonate. Complete removal of volatile salt was accomplished by re-dissolving the solid in methanol. These procedures created an amine on the anomeric carbon for future conjugation with PGA.

To conjugate aminated Di-mannose to PGA, the substrate was dissolved in water to 30 mg m/L, then sonicated for 10 min Ethyl-N′-(3-dimethylaminopropyl) carbodiimide•HCl in water (4 mg/mL, 30 equiv.) was added with mixing at RT for 4 min N-hydroxysulfosuccinimide in water (30 mg/mL, 35 equiv.) was incubated with the PGA/EDC solution for 1 min Aminated Di-mannose in phosphate-buffered saline (PBS) was combined with the resulting activated PGA in a 44:1 molar ratio and mixed at RT for 6 h. Excess reagents were removed by dialysis against water for 24 h.

### mRNA synthesis

Codon-optimized mRNA for eGFP, IRF5, and IKK (TriLink Biotechnologies) were capped with the Anti-Reverse Cap Analog 3′-O-Me-m7G(5′)ppp(5′)G (ARCA), and fully substituted with the modified ribonucleotides pseudouridine (Ψ) and 5-methylcytidine (m5C).

### Nanoparticle preparation

IRF5 and IKKβ mRNAs were combined at a 3:1 (w:w) ratio and diluted to 100 μg/mL in 25 mM sodium acetate (NaOAc) buffer (pH = 5.2). PbEs)−447 (PbAE-447) polymer in DMSO (prepared as described above) was diluted from 100 μg/μL to 6 μg/μL, also in NaOAc buffer. To form the nanoparticles, PbAE-447 polymers were added to the mRNA at a ratio of 60:1 (w:w) and vortexed immediately for 15 s at a medium speed, then the mixture was incubated at RT for 5 min to allow the formation of PbAE-mRNA polyplexes. In the next step, 100 μg/mL PGA/Di-mannose in NaOAc buffer was added to the polyplexes solution, vortexed for 15 s at medium speed, and incubated for 5 min at room temperature. In this process, PGA/Di-mannose coated the surfaces of PbAE-mRNA polyplexes to form the final NPs. For long-term storage, D-sucrose (60 mg/mL) was added to the NP solutions as a cryoprotectant. The nanoparticles were snap-frozen in dry ice, then lyophilized. The dried NPs were stored at −20 °C or −80 °C until use. For in vivo experiments, lyophilized NPs were re-suspended in water at a 1:20 (w:v) ratio.

### Characterization of nanoparticle size distribution and ζ-potential

The physiochemical properties of NPs (including hydrodynamic radius, polydispersity, ζ-potential, and stability) were characterized using a Zetapals instrument (Brookhaven Instrument Corporation) at 25 °C. To measure the hydrodynamic radius and polydispersity based on dynamic light scattering, NPs were diluted fivefold into 25 mM NaOAc (pH = 5.2). To measure the ζ-potential, NPs were diluted 10-fold in 10 mM PBS (pH = 7.0). To assess the stability of NPs, freshly prepared particles were diluted in 10 mM PBS buffer (pH = 7.4). The hydrodynamic radius and polydispersity of NPs were measured every 10 min for 5 h, and their sizes and particle concentrations were derived from Particle Tracking Analysis using a Nanosite 300 instrument (Malvern). Freshly made NPs (25 μL containing 0.83 μg of mRNA) were deposited on glow discharge-treated 200 mesh carbon/Formvar-coated copper grids. After 30 s, the grids were treated sequentially with 50% Karnovsky’s fixative, 0.1 M cacodylate buffer, dH_2_O, then 1% (w/v) uranyl acetate. Samples were imaged with a JEOL JEM-1400 transmission electron microscope operating at 120 kV (JEOL USA).

### Bone marrow-derived macrophages (BMDMs) and other cell lines

To prepare BMDMs, bone marrow progenitor cells were harvested from mouse femurs following established protocols^71^. These cells were cultured in complete medium [DMEM supplemented with 4.5 g/L D-glucose, L-glutamine, 10% heat-inactivated FBS, 100 U/mL penicillin and 100 μg/mL, Glutamax 50 mL/500 mL, supplemented with 20 ng/mL M-CSF (Peprotech, cat#315–02)] at a seeding density of 0.5–1.0 e6/ml. Cells were allowed to differentiate into BMDMs ex vivo for 7 days under 5% CO_2_ at 37 °C. Next, they were conditioned with macrophage-conditioned medium [macrophage complete medium supplemented with 20 ng/mL MPLA (Sigma, cat#L6895) or 20 ng/mL IL4 (eBioscience, cat# 34–8041)]. BMDMs were used between 7–21 days ex vivo. The murine ovarian cancer cell line ID8, a gift from Dr. Katherine Roby (University of Kansas Medical Center, Kansas City, KS), was cultured in DMEM supplemented with 10% FBS, 100 U/mL penicillin, 5 μg/mL insulin, 5 μg/mL transferrin, and 5 ng/mL sodium selenite (all Sigma-Aldrich). To generate the more aggressive VEGF-expressing ID8 strain, we transfected ID8 tumor cells with the pUNO1 plasmid (InvivoGen) encoding murine VEGF along with the blasticidin-resistance gene. To obtain stable transfectants, tumor cells were cultured in complete medium containing 10 μg/mL blasticidin (InvivoGen) for 3 weeks. The B16F10 melanoma cell line (American Type Culture Collection) was cultured in complete RPMI 1640 medium with 10% FBS, 100 U/mL penicillin, 2 mM/L-glutamine, 1.5 g/L sodium bicarbonate, 4.5 g/L glucose, 10 mM HEPES, 1.0 mM sodium pyruvate, and 0.05 mM 2-mercaptoethanol. For in vivo bioluminescent imaging, both ID8-VEGF and B16F10 cell lines were retrovirally transduced with firefly luciferase. The DF-1 cell line carrying RACS-PDGFβ or RCAS-cre retrovirus was cultured in complete medium supplemented with 10% FBS and 100 U/mL penicillin under 5% CO_2_ at 39 °C_._

### mRNA transfection of BMDMs

One day prior to transfection, BMDMs were reseeded on 24-well plates in macrophage complete medium at a concentration of 250,000/well. Before transfection, the complete medium was replaced with 300 µL unsupplemented DMEM. To transfect these cells, NPs containing 2 µg mRNA were added into the base medium and co-cultured with the BMDMs at 37 °C. After 1 h, medium containing NPs was removed, and the cells were cultured an additional 24 h before evaluation of transfection efficiency and cell viability.

### Transfection of BMDMs for macrophage signature gene analysis

BMDMs were reseeded on 24-well plates in conditioned medium 24 h prior to transfection, allowing transformation of the cells into their phenotypes. M2-like macrophages were then exposed to either IRF5/IKKβ NPs carrying 25% eGFP mRNA as a reporter, or eGFP NPs (control) containing 2 µg mRNA, following the transfection protocol described above. After 24 h, the top 10% of highly transfected BMDMs (as measured by eGFP expression) were sorted at 24 h after transfection and were re-challenged in low-dose (10 ng/mL) IL4 medium for another 48 h before RNA isolation. RNAs extracted from these cells were compared to those from standard M1- or M2-like macrophages so we could identify signature genes associated with IRF5-NP treatment.

### RNA isolation and preparation

To harvest RNAs, BMDMs were lysed in Trizol reagent (Ambion), and total RNAs were extracted and purified using RNeasy® Plus Universal Mini-Kits (QIAGEN) following the manufacturer’s instructions. Sample RNA was quantified using a NanoDrop Microvolume Spectrophotometer (Thermo Fisher) and then subjected to quality control performed by the *FHCRC Genomics Shared Resource* with an Agilent 4200 TapeStation analyzer (Agilent).

### IRF5 protein ELISA

Following nanoparticle transfection, macrophage cell pellets were subjected to total protein extraction using RIPA Lysis and Extraction Buffer supplemented with Halt Protease Inhibitor Cocktail and Halt Phosphatase Inhibitor Cocktail (Thermo Fisher Scientific), following the manufacturer's protocol. After the protein concentration in each sample was quantified via a BCA assay, the IRF5 concentration from each sample was evaluated using the ELISA assay (LS-F21481, LSBio, WA). The IRF5 concentrations measured by ELSA were normalized by the total protein concentration.

### Macrophage signature gene analysis using NanoString technology

Gene expression values from stimulated BMDM cultures were measured using the nCounter® Myeloid Innate Immunity Panel (NanoString Technology), which analyzes 770 genes occurring in 19 different pathways and processes them across seven different myeloid cell types. The samples were tested using an nCounter Analysis System (NanoString Technologies). Raw data were processed and checked for quality using the R/Bioconductor NanoStringQCPro software package^72^. Expression values were normalized to the geometric mean of housekeeping genes and log2-transformed using nSolver 4.0 software (NanoString Technologies). False Discovery Rates for ratio data were calculated from the *p*-values returned by the *t*-tests using the Benjamini-Yekutieli method.

### Flow cytometry and cell sorting

Cells obtained from spleen, blood, peritoneal lavage, and bronchoalveolar lavage were analyzed by flow cytometry with myeloid and lymphoid immunophenotyping panels using the anti-mouse antibody probes listed in Supplementary Table 1. Primary antibodies applied in the flow cytometry analysis are listed as follows: CD45 (eBioscience catalog # 48–0451, 1:800), MHC I-A/I-E (Biolegend catalog # 107622, 1:400), CD11b (BD Biosciences catalog # 557657, 1:200), CD11c (BD Biosciences catalog # 5624547, 1:200), Ly6C (eBioscience catalog # 45–4932, 1:200), Ly6G (Biolegend catalog # 127624, 1:200), CD206 (Biolegend catalog # 141732, 1:200), CD335 (Nkp46) (BD Biosciences catalog # 565085, 1:800), CD4 (Biolegend catalog # 100540, 1:400), CD44 (BD Biosciences catalog # 562464, 1:400), CD49B (BD Biosciences catalog # 740250, 1:200), CD62L (Biolegend catalog # 104428, 1:200), CD8 (Biolegend catalog # 100712, 1:400), TCR- β chain (Biolegend catalog # 127908, 1:400), F4/80 (eBioscience catalog # 12–4801, 1:400). Data were collected using a BD LSRFortessa analyzer running FACSDIVA software (Beckton Dickinson). CD11b+ and F4/80+ peritoneal macrophages were sorted using BD FACS ARIA II. All collected data were analyzed using FlowJo 10.0 software.

### Cytokine analysis

Cytokine levels were evaluated using a Luminex 200 system (Luminex) at the *FHCRC Immune Monitoring Shared Resource* center. For ex vivo studies, cell culture supernatant was collected for the measurement of IL-6, IL-12p70, INFγ, and TNFα concentrations. For in vivo studies, plasma concentration of GM-CSF, INFγ, IL-12p70, IL-2, IL-6, and TNFα were measured. IL-1β cytokine levels were measured using the Invitrogen ELISA kit (REF#88–7013).

### Histology

Immunofluorescence, immunohistochemistry (IHC), and hematoxylin and eosin (H&E) analysis were performed on mouse intestinal mesentery tissue. For all histopathology analyses, tissues were fixed in 4% neutral buffer formalin before further processing. Four-micron sections were cut and stained with the Leica Bond Rx (Leica Biosystems, Buffalo Grove, IL). For IHC DAB staining, slides were pretreated with Leica Bond Epitope Retrieval Solution for 20 min Endogenous peroxidase was blocked with Leica peroxide block for 5 min A TCT protein block was applied for 10 min (0.05 M Tris, 0.15 M NaCl, 0.25% Casein, 0.1% Tween 20, pH 7.6). Primary antibody was applied to the tissue for 60 min The antibody was then detected using a specific polymer and staining was visualized with Bond Polymer Refine Detection DAB (Leica Biosystems catalog# D59800); a hematoxylin counterstain was also used (Leica). Primary antibodies applied in the immunofluorescence and IHC DAB analysis are listed as follows: Ly6B.2 (Bio-Rad catalog # MCA771GA, 1:7500), F4/80 (Cell Signaling catalog # 770765, 1:6000), CD4 (eBioscience catalog # 14–976–32, 1:4000), CD8 (eBioscience catalog # 14–0808–82, 1:4000), Cytokeratin 8/18 (University of Iowa Hybridoma Bank; catalog # TROMA-1, 1:100). DAPI (Sigma Catalog 8417–10MG) was used at 5 µg/ml in PBS.

A Perkin Elmer Vectra 3.0 Automated Imaging Platform was used to acquire the fluorescence images of intestinal mesentery slides under ×20 magnification. These images were analyzed using HALO Image Analysis Modules. The Cytonuclear for FL function was applied to calculate %CD4+ cells, %CD8+ cells and %F4/80+ %Ly6B.2+ cells among all cells in the tumor region. For each sample group, five tissue slides prepared from three mice were analyzed to obtain statistically significant data.

### qRT-PCR analysis

Gene expression levels were determined by qRT-PCR. To measure selected macrophage signature genes (SerpinB2, Retnla, Ccl5, Ccl11, codon-optimized IRF5, endogenous IRF5, and housekeeping GAPD genes), total RNA was isolated with RNeasy mini-columns (Qiagen) according to the manufacturer’s instructions. cDNA was synthesized using a qScript cDNA Synthesis Kit (Quanta). For each sample, qRT-PCR was performed in triplicate via PerfeCTa qPCR SuperMix Low ROX (Quanta) using gene-specific probes from the Roche’s Universal Probe Library (UPL) and PCR primers optimized by ProbeFinder (Roche): SerpinB2, UPL -049, F-ACTGGGGCAGTTATGACAGG, R-GATGATCGGCCACAAACTG; Retnla, UPL-078, F-TTGTTCCCTTCTCATCTGCAT, R-CCTTGACCTTATTCTCCACGA; Ccl5, UPL-105, F-CCTACTCCCACTCGGTCCT, R-CTGATTTCTTGGGTTTGCTGT; Ccl11, UPL-018, F-AGAGCTCCACAGCGCTTC, R- CAGCACCTGGGAGGTGAA; codon-optimized IRF5, UPL-022, F-TCTTAAAGACCACATGGTAGAACAGT, R-AGCTGCTGTTGGGATTGC; endogenous IRF5, UPL-011, F-GCTGTGCCCTTAACAAAAGC, R-GGCTGAGGTGGCA TGTCT. Signature gene mRNA levels were normalized based on amplification of GAPD, UPL-060, F-AGCCACATCGCTCAGACAC and R-GCCCAATACGACCAAATCC. All qRT-PCR reactions were performed using Quant Studio5 RT-PCR machines running QuantStudio6 software (Applied Biosystems). In cases when the amplification plot did not cross the threshold and no Ct value was obtained (“undetermined”), a Ct value equal to the highest cycle number of in the assay (40 cycles) was used for comparisons of relative expression.

### Mice and in vivo tumor models

Except for the brain tumor model-related experiments, the mice used in these experiments were obtained from Jackson Laboratory; the others were bred and housed in the FHCRC animal facility. All of the mice were used in the context of a protocol approved by the center’s *Institutional Animal Care and Use Committee*. To model ovarian tumors, 5 × 10^6^ vascular epithelial growth factor (VEGFP)-expressing ID8 cells were injected intraperitoneally (i.p.) into 4- to 6-week-old female albino B6 (C57BL/6J-Tyr<c-2J>) mice and allowed to establish for 2 weeks. For survival studies, the animals were treated i.p. with IRF5 NPs/eGFP NPs carrying 50 μg mRNA (two doses per week for 9 weeks, or until health conditions reached euthanizing requirements). For mechanism studies, we used the treatments for either 1, 2, or 3 weeks, followed by euthanization at 48 h following the last dose. Peritoneal lavage was performed to collect the peritoneal cells. To compare the efficacy of IRF5/IKKβ NPs with status quo macrophage targeting therapies, one group of mice received treatment with IRF5/IKKβ NPs carrying 50 μg mRNA for 3 weeks with 2 doses per week; the second received oral gavage of 15 mg/kg PI3Kγ inhibitor IPI-594 (MedKoo Biosciences Inc) formulated in vehicle (5% 1-methyl-2-pyrrolidinone in polyethylene glycol 400) daily for 3 weeks; the third group received i.p. injection of 30 mg/kg CSF1R inhibitor Pexidartinib (PLX3397, MedKoo Biosciences Inc) formulated in the same vehicle daily for 3 weeks.

To model metastatic lung cancer, 2.5 × 10^4^ B16F10 cells transduced with F-luc and suspended in 200 μL RPMI medium were injected into 4- to 6-week-old female albino B6 (C57BL/6J-Tyr<c-2J>) mice (Jackson Laboratories) and allowed to establish for 1 week. For survival studies, mice were treated retro-orbitally with (or without) IRF5/IKKβ or eGFP NPs carrying 30 μg mRNA suspended in PBS. Mice were treated with 3 doses/wk for 3 weeks or until health conditions reached euthanizing requirements. For mechanism studies, the mice received the same treatments for 2 weeks. Bronchoalveolar lavage was performed to collect alveolar cells for analysis.

Mice bearing glioma were generated following published protocols^49^. Avian DF-1 cells producing RCAS-PDGFβ and RCAS-cre retroviruses were injected intracranially into both brain hemispheres (coordinates: 1 mm caudal from bregma, 2 mm lateral, depth of 2 mm from the dural surface) of Nestin-tv-a/Ink4a-arf−/−; Pten−/− mice (C57BL/6) between 4–6 weeks of age. Tumors were allowed to establish for 2 weeks. At day 15, mice received 10Gy radiation to one hemisphere, while the unirradiated hemisphere was shielded with lead. The next day, mice received retro-orbital injections of IRF5/IKKβ NPs carrying 30 µg mRNA (3 doses/wk for 3 weeks), or were assigned to the PBS control group.

### In vivo bioluminescence imaging

D-Luciferin (Xenogen) in PBS (15 mg/mL) was used as a substrate for firefly luciferase imaging. Bioluminescence images were collected with a Xenogen IVIS Spectrum Imaging System (Xenogen). Mice were anesthetized with 2% isoflurane (Forane, Baxter Healthcare) before and during imaging. For ID8-VEGF ovarian tumors, each mouse was injected i.p. with 300 µg of D-Luciferin, and images were collected 10 min later. For B16F10 lung metastatic tumors, mice were injected i.p. with 3 mg of D-Luciferin, and images were collected 15 min afterwards. For brain tumor models, the mice received retro-orbital injection of 75 mg/kg body weight D-Luciferin, and images were collected 4 min later. Acquisition times ranged from 10 s to 5 min.

### Biodistribution analysis

To determine the biodistribution of IRF5 NPs in the ID8-VEGF ovarian tumor model, mice in 7–8 groups received an i.p. or retro-orbital dose of NPs carrying 50 µg mRNA. Twenty-four hours after injection, whole blood was collected and mice were euthanized with CO_2_ to retrieve organs (liver, spleen, lung, kidney, heart, intestine, pancreases, and diaphragm). All tissues were stabilized with RNA*later*, then frozen on dry ice. The codon-optimized IRF5 mRNA levels in each organ were measured using RT-qPCR.

### Toxicity analysis

To measure potential in vivo toxicities of repeatedly injecting macrophage-targeting NPs, we injected mice (5/group) intraperitoneally or intravenously with six sequential doses of IRF5/IKKβ or eGFP mRNA NPs. Controls received no treatment. Twenty-four hours after the final infusion, mice were anesthetized and blood was collected by retro-orbital bleed to determine the complete blood counts. Blood was also collected for serum chemistry and cytokine profile analyses (performed by Phoenix Central Laboratories, Mukilteo, WA). Animals were then euthanized with CO_2_ to retrieve organs, which were washed with deionized water before fixation in 4% paraformaldehyde. The tissues were processed routinely, and sections were stained with H&E. The specimens were interpreted by board-certified staff pathologists, in a blinded fashion.

### Cytokine assays

Cytokine levels were evaluated using a Luminex 200 system (Luminex) at the *FHCRC Immune Monitoring Shared Resources*. For ex vivo studies, cell culture supernatant was collected for the measurement of IL-6, IL12p70, INFγ, and TNFα concentrations. For in vivo studies, we measured plasma concentrations of IL-6, TNFα, and IL-1β.

### Statistical analysis

The statistical significance of observed differences were analyzed using the unpaired, two-tailed Student’s *t* test or the unpaired, two-tailed one-way ANOVA test. The *P* values for each measurement are listed in the figures or figure legends. We characterized survival data using the Log-rank test. All statistical analyses were performed either using GraphPad Prism software version 6.0 or R software.

### Study approval

The care and use of mice in this study was approved by the Institutional Animal Care & Use Committee (IACUC) at the Fred Hutchinson Cancer Research Center, and was in compliance with all relevant ethical regulations for animal testing and research (Assurance #A3226–01, IACUC Protocol Number 50782).

### Reporting summary

Further information on research design is available in the Nature Research Reporting Summary linked to this article.

## Supplementary information

Supplementary Information

Reporting Summary

## Data Availability

The Raw and processed data from the NanoString gene expression assays data have been deposited in the NCBI's Gene Expression Omnibus database under the accession code GSE120254 and GSE129498. All the other data supporting the findings of this study are available within the article and its supplementary information files and directly from M. Stephan upon reasonable request. A reporting summary for this article is available as a Supplementary Information file.
