# Supplementary material for: Genetic programming of macrophages to perform anti-tumor functions using targeted mRNA nanocarriers
Source: Nat Commun. 2019 Sep 3;10:3974. doi: 10.1038/s41467-019-11911-5 (PMC6722139; doi:10.1038/s41467-019-11911-5)
Supplement: Supplementary file 1 — Supplementary Information [file 41467_2019_11911_MOESM1_ESM.pdf]

Supporting Online Material for

Genetic programming of macrophages to perform anti-tumor functions using targeted mRNA nanocarriers

F. Zhang[1], N. N. Parayath[1], C. I. Ene[2], S. B. Stephan[1], A. L. Koehne[3], M. E. Coon[1], E. C. Holland[2,4,5] & M. T. Stephan[‡1,6,7]

[1]Clinical Research Division, Fred Hutchinson Cancer Research Center, Seattle, Washington 98109, USA.

[2]Department of Neurological Surgery, University of Washington School of Medicine, Seattle, Washington, USA.

[3]Comparative Pathology, Fred Hutchinson Cancer Research Center, Seattle, Washington 98109, USA.

[4]Human Biology Division, Fred Hutchinson Cancer Research Center, Seattle, Washington 98109, USA.

[5]Alvord Brain Tumor Center, University of Washington, Seattle, Washington 98195, USA.

[6]Department of Bioengineering and Molecular Engineering & Sciences Institute, University of Washington, Seattle, Washington 98109, USA.

[7]Department of Medicine, Division of Medical Oncology, University of Washington, Seattle, Washington 98195, USA.

[‡]Correspondence should be addressed to M.T.S. (mstephan@fredhutch.org)

This PDF file includes:

Supplementary Figs. 1-5 with figure captions
Supplementary Table 1

a

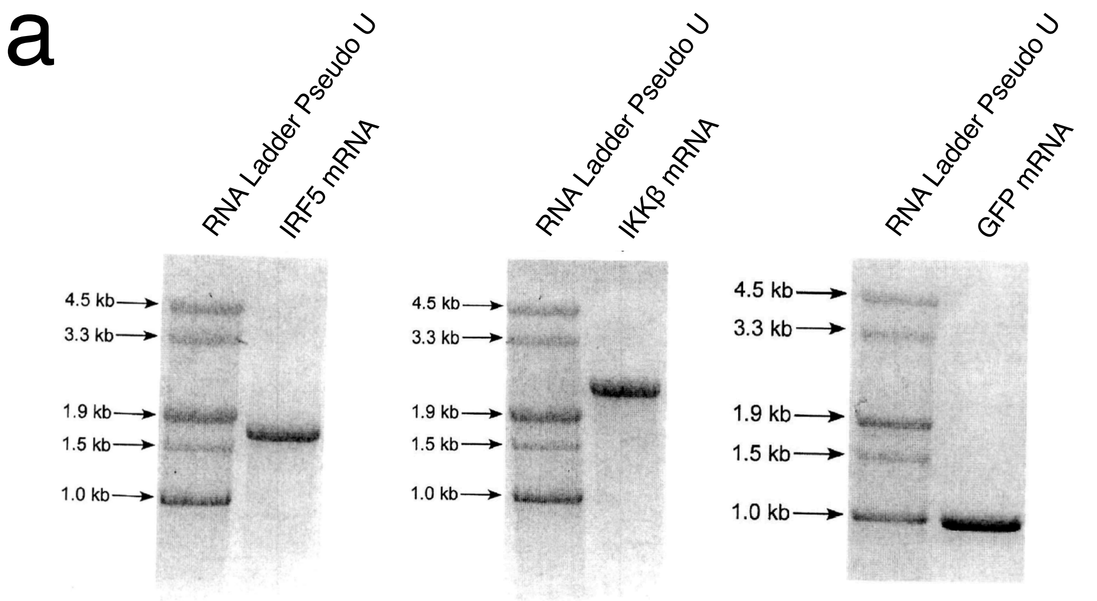

b

Sequencing result by PacBio:
mIRF5 and mIKKb produced by TriLink were sequenced by Pacific Biosciences Inc. using their SMRT sequencing technology, under SMRT Link 5.1 Iso-Seq Pipeline. The sequencing experiment generated 382 (mIRF5) and 113 (mIKKb) polished high-quality isoforms.

Screening of final mIRF5 and mIKKb isoform candidates:
To identify the isoforms with the highest sequence alignment with the original mIRF5 and mIKKb sequences (this refers to the sequences we submitted to TriLink, therefore do not include ARCA cap, PolyA tails, and other base modifications), Nucleotide BLAST from NIH was used to screen all polished high-quality isoforms.

For mIRF5 isoforms screening, we found 37 out 382 isoforms that has 100% alignment rates with the original sequence. Among these 37 isoforms, 17 have repeated match ≥2 (meaning dimmers or trimers). After excluding these 17 isoforms, we got 20 isoforms with 100% alignments with the original IRF5 sequence (sequence length = 1494). These 20 isoforms have sequence length between 1639 – 1965, which are in the similar range with the length of mIRF5 sequence reported by TriLink (sequence length = 1770).

For mIKKb isoforms screening, we found 10 out 113 isoforms that has 100% alignment rates with the original sequence. Among these 10 isoforms, 5 have repeated match ≥2 (meaning dimmers or trimers). After excluding these 5 isoforms, we got 5 isoforms with 100% alignments with the original IKKb sequence (sequence length = 2150). These 5 isoforms have sequence length between 2305 – 2404, which are in the similar range with the length of mIKKb sequence reported by TriLink (sequence length = 2440).

All screened final mIRF5 and mIKKb sequence isoforms were uploaded as **Supplementary Data 1** and **2,** respectively.

c

Calibration curve after third order polynomial fitting: NT = 0.1023*T^3 – 5.093*T^2 + 89.32*T^1 – 574.6*T^0
(NT= nucleotide; T=time (min))

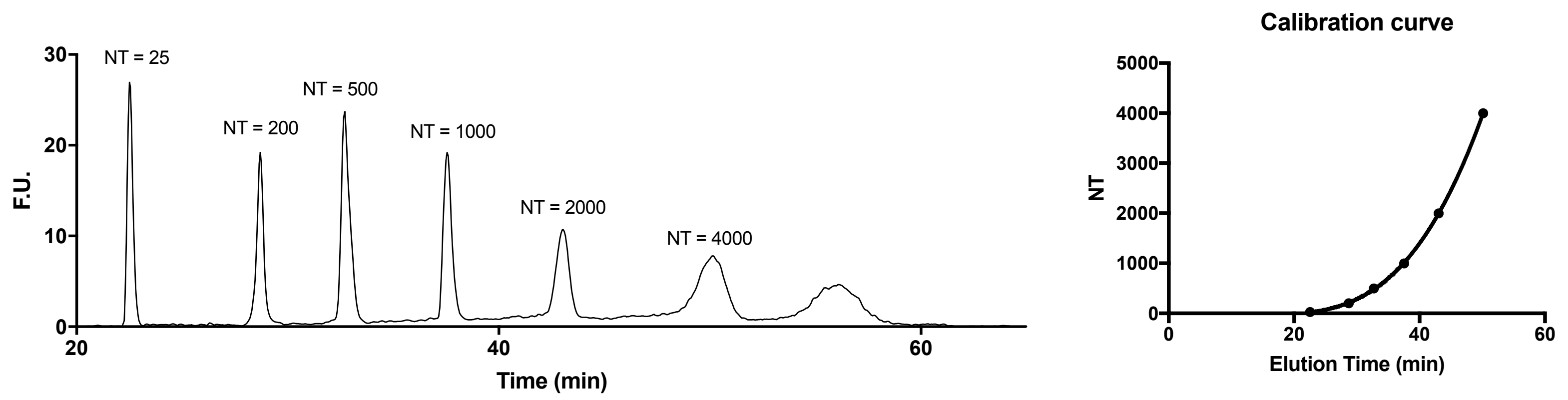

d

Mean purity (based on 3 independently manufactured batches)

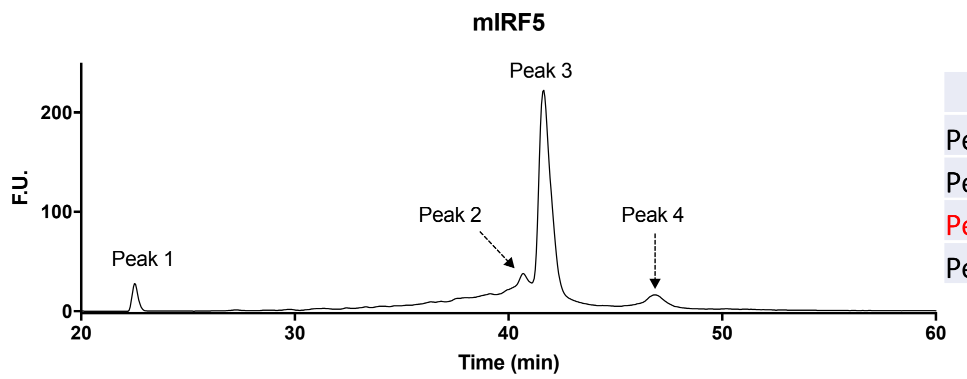

	NT	Mass %
Peak 1	22	4.15
Peak 2	1521	9.33
Peak 3	1702	75.24
Peak 4	3067	11.29

78.21% ± 3.3%

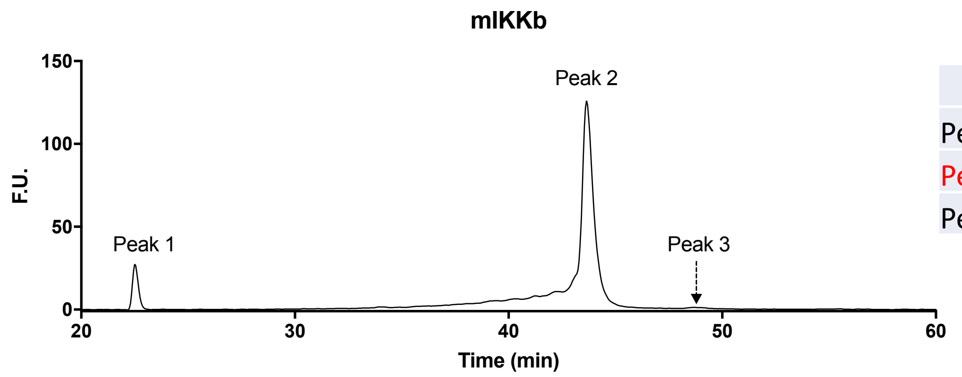

	NT	Mass %
Peak 1	22	9.11
Peak 2	2128	89.32
Peak 3	3895	1.57

91.5% ± 2.02%

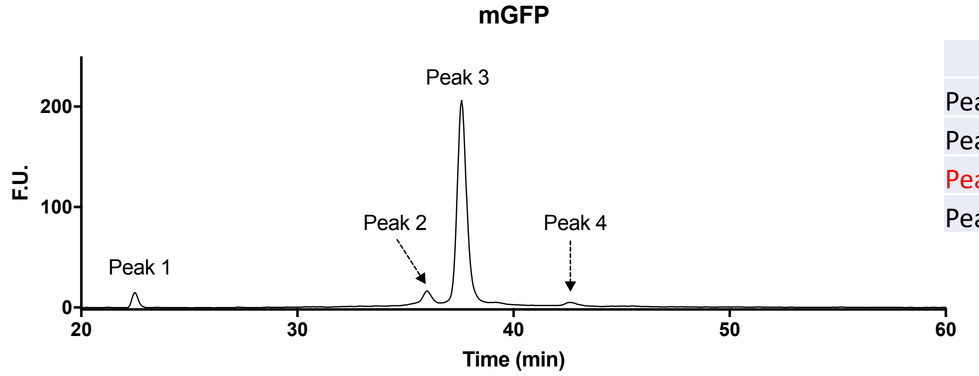

	NT	Mass %
Peak 1	23	3.54
Peak 2	818	8.40
Peak 3	1027	80.81
Peak 4	1910	7.24

82.3% ±3.4%

Supplementary Fig. 1: Integrity and purity of IVT mRNA used in our experiments. (**a**) Representative agarose gel electrophoresis of IVT mRNA encoding IRF5 (left panel), IKKβ (middle panel), and GFP (right panel). The expected size for mouse IRF5 mRNA is 1,770 bp, which includes the actual coding sequence (1,494 bp + the 5'UTR and the polyA track). The expected size for mouse IKKβ mRNA is 2,430 bp (2,154 bp + 5'UTR + polyA track), and that for the GFP control mRNA is 1,027 bp (996 bp + 5'UTR + polyA track). (**b**) Single Molecule Real-Time (SMRT) sequencing of IVT mRNA constructs. (**c**) Bioanalyzer electropherogram calibration curve. Representative curves of IVT mRNA encoding murine IRF5, IKKβ, and GFP are shown in (**d**). Based on the calibration curve, the major fractions (peak 3 for IRF5 and GFP and peak 2 for IKKβ) represent the relevant single-stranded mRNA. The last fractions (peak 4 for IRF5 and GFP and peak 3 for IKKβ) represent impurities from double-stranded mRNA. Mean purities (based on three independently manufactured mRNA batches) are shown on the right.

a

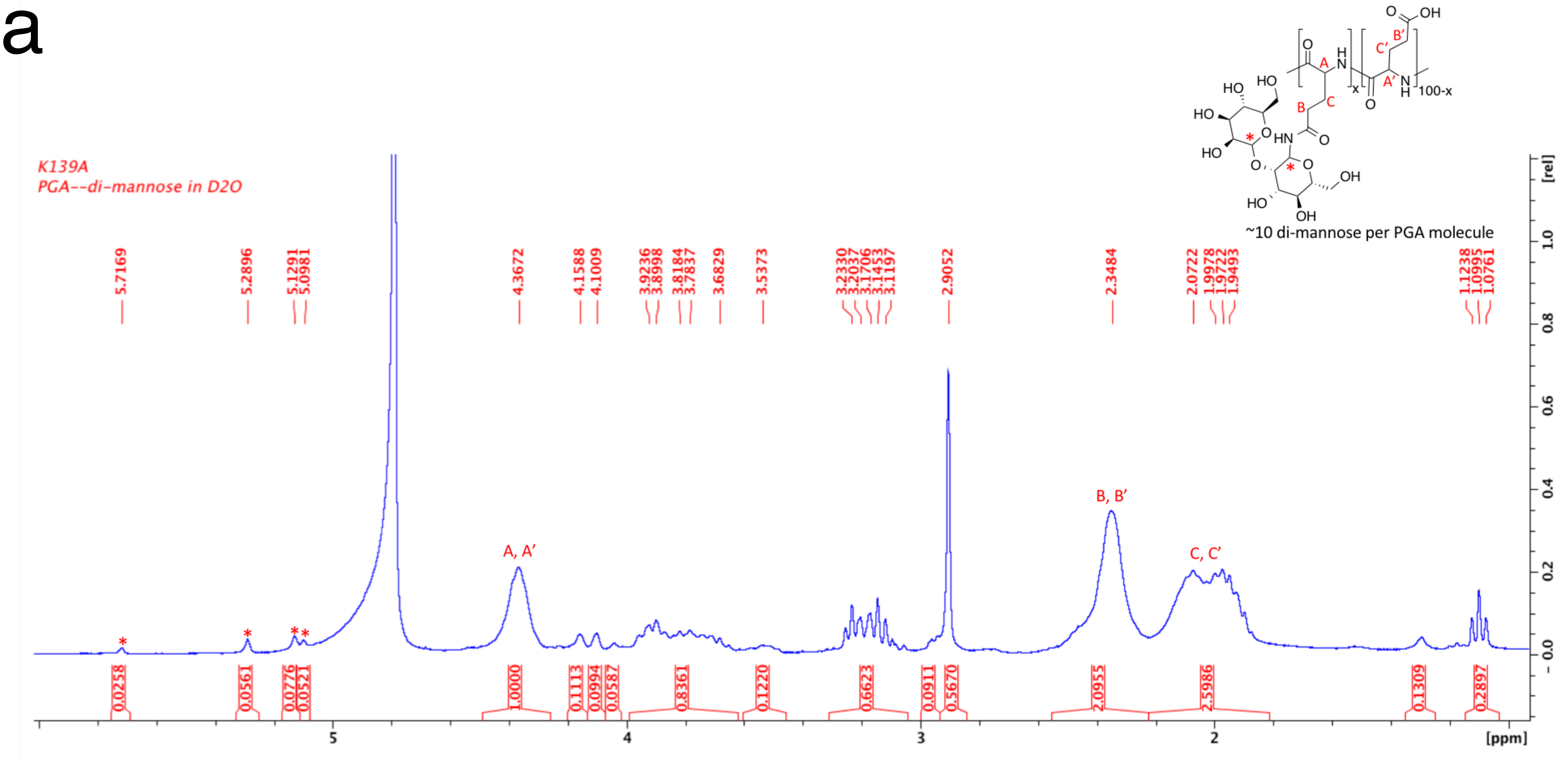

Supplementary Fig. 2: (a) Efficient covalent coupling of Di-mannose to PGA polymer using EDC/NHS bioconjugation. Representative 1H NMR spectroscopy of polyglutamic acid (PGA) following functionalization with Di-mannose. We routinely used 1H NMR spectroscopy in D_2O (Bruker Avance, 300 Mhz) as a quality control for successful conjugation of Di-mannose to PGA. Peaks corresponding to the anomeric protons of Di-mannose were observed between 5.1 and 5.7 ppm. The ratio of Di-mannose to PGA was roughly estimated based on integration of the anomeric proton peaks (*) and the PGA C-H peaks (A and A').

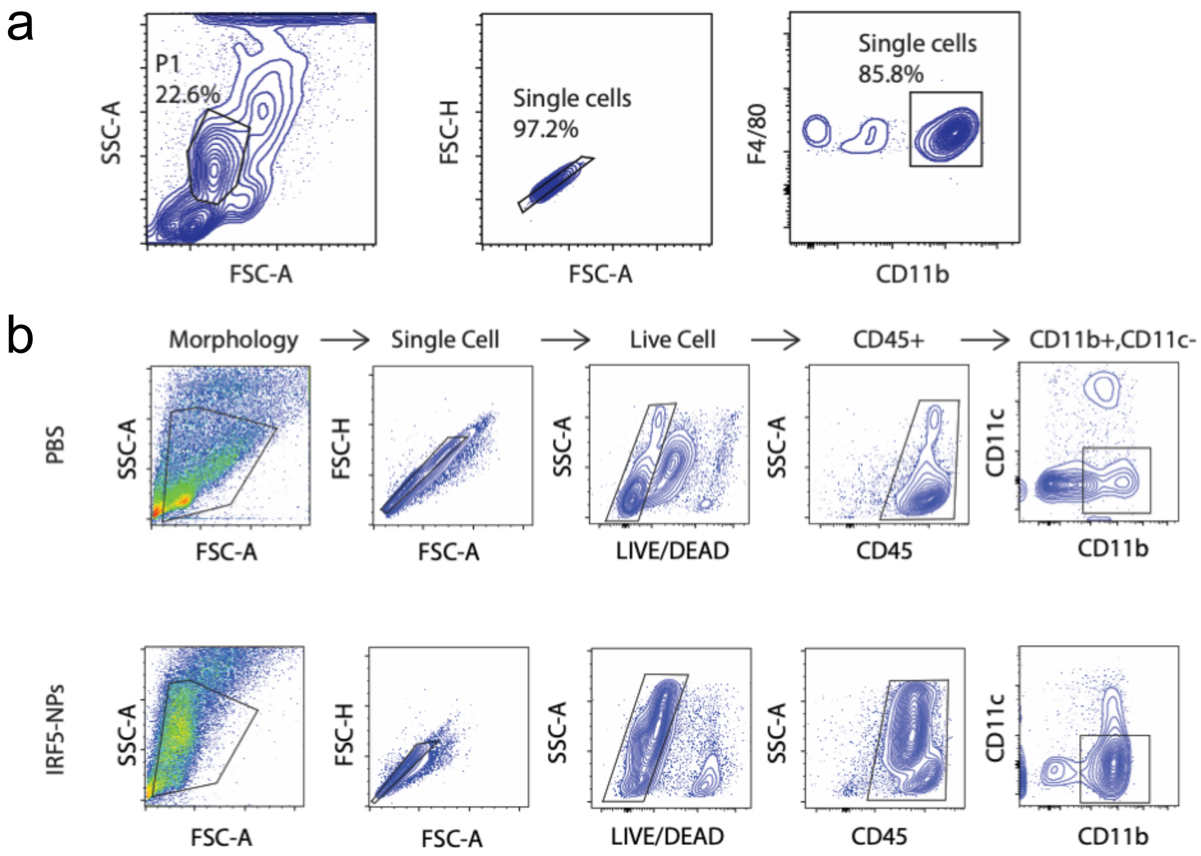

Supplementary Fig. 3: Gating Strategies for multicolor flow cytometry analysis and cell sorting. **(a)** FACS sequential gating strategies for isolating CD11b+ and F4/80+ peritoneal macrophages for **Fig 3 i & j,** *ex vivo* cytokine release study and qRT-PCR analysis of selected macrophage signature genes; **(b)** FACS sequential gating strategies for isolating CD45+, CD11b+, CD11c- peritoneal macrophages for **Supplementary Fig. 4** nanoString's gene expression analysis.

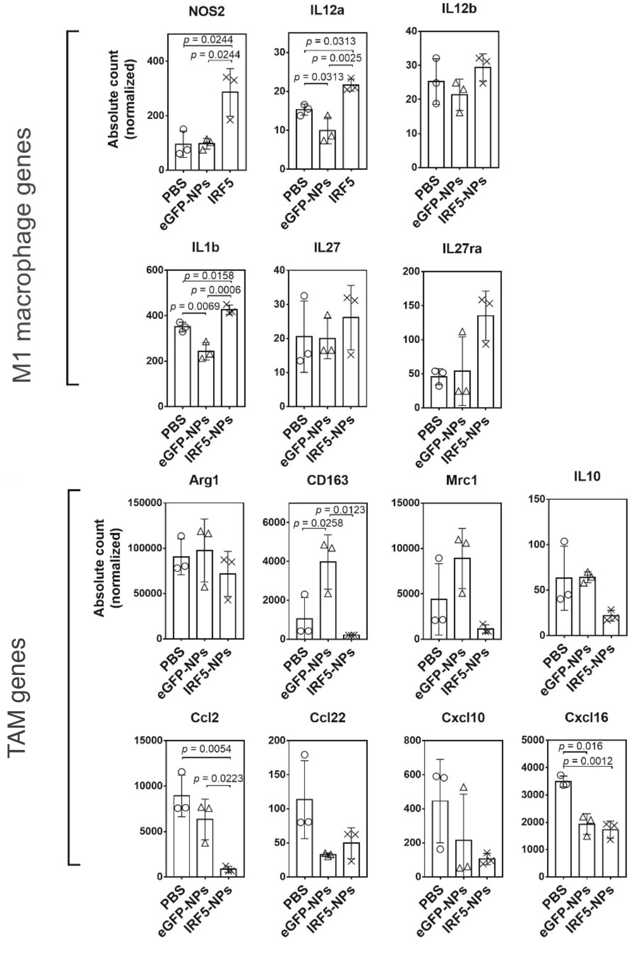

Supplementary Fig. 4. Effects of IRF5/IKKb nanoparticles on inflammation-related genes among the 770 genes analyzed in NanoString's nCounter® Mouse Myeloid Innate Immunity Panel v2 (the complete gene list and expressions are available on NCBI's GEO repository, Accession number GSE129498). Nanoparticle transfection upregulated the expression of genes associated with M1-like inflammatory macrophages such as NOS2 (199% increase), IL12a (41% increase), IL12b (16% increase), IL1b (22% increase), IL27 (27% increase), IL27a (194% increase), while downregulating the expression of genes associated TAMs such as Arg1 (21% decrease), CD163 (80% decrease), Mrc1 (or CD206) (74% decrease), IL10 (65% decrease), Ccl2 (90% decrease), Ccl22 (56% decrease), Cxcl10 (76% decrease), and Cxcl16 (51% decrease). Specifically, genes that were significantly changed between IRF5-NPs treated and PBS treated groups were NOS2 (P=0.0224), IL12a (P=0.0313), IL1b (P=0.0158), Ccl2 (P=0.0054), Cxcl16 (P=0.0016).

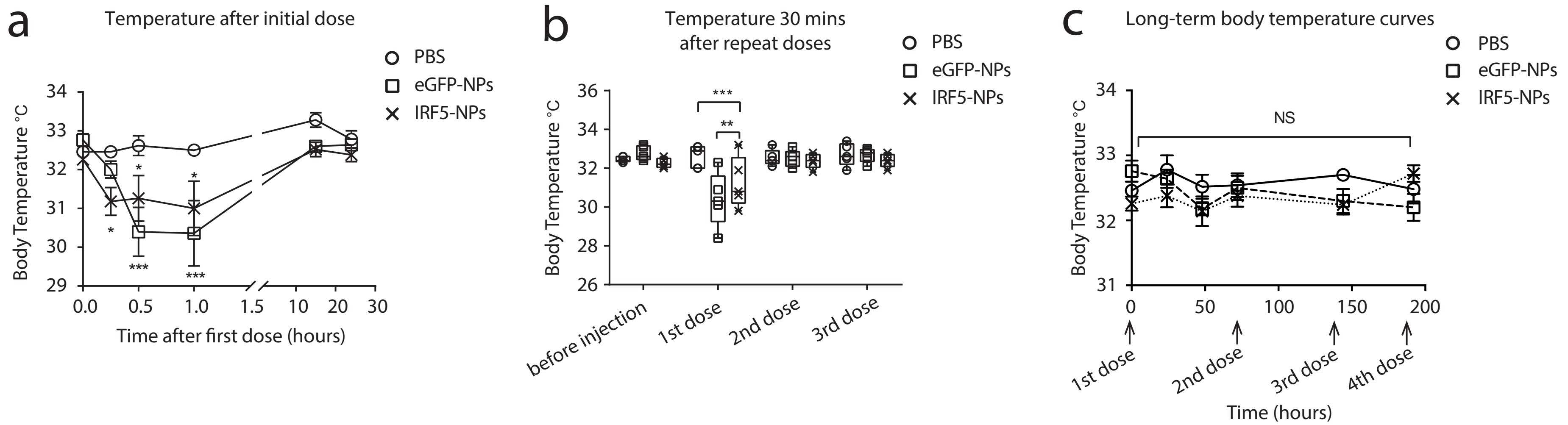

Supplementary Fig. 5: Programming proinflammatory macrophages with IRF5/IKKβ nanoparticles does not trigger fever. Body temperatures of C57BL/6 mice were serially monitored following repeated intravenous administration of nanoparticles loaded with mRNA (50 μg/dose) encoding IRF5/IKKβ or control GFP. A third group of mice was injected with PBS. Body temperatures were measured using an infrared thermometer 0 h, 15 min, 30 min, 1 h, 15 h and 24 h after each treatment. (**a**) Temperature changes following the initial nanoparticle dose. Each point represents the mean ± s.e.m. We noticed a significant ~1-2 °C drop in body temperature 30 min after treatment (with both eGFP-NPs and IRF5/IKKβ-NPs). Following all subsequent nanoparticle injections, body temperature remained normal, as shown in (**b**) and (**c**). Data are from ten mice per treatment condition pooled from two independent experiments.

Supplementary Table 1. List of primary antibodies and other staining reagent used for flow cytometry, flow-assisted cell sorting analysis, immunohistochemistry analysis and immunofluorescence analysis.

List of antibodies and other staining reagent used for flow cytometry and flow-assisted cell sorting analysis.

Antibody Specificity	Clone	Isotype	Dilution	Dye	Supplier	Catalog#
**Myeloid Panel**						
CD45	30-F11	Rat IgG2b, k	1:800	eFluor 450	eBioscience	48-0451
MHC I-A/I-E	M5/114.15.2	Rat IgG2b, k	1:400	AlexaFluor700	Biolegend	107622
CD11b	M1/70	Rat IgG2b, k	1:200	APC	BD Biosciences	557657
CD11C	N418	Arm Ham IgG	1:200	PE-CF594	BD Biosciences	562454
Ly6C	HK1.4	Rat IgG2a, k	1:200	PerCP-Cy5.5	eBioscience	45-4932
Ly6G	1A8	Rat IgG2a, k	1:200	APC-Cy7	Biolegend	127624
CD206	C0682C2	Rat IgG2a, k	1:200	PE-Dazzle 594	Biolegend	141732
Live/Dead Fixable Green	-	-	1:800	FITC	Life Technologies	L23101
**Lymphoid Panel**						
CD45	30-F11	Rat IgG2b, k	1:800	eFluor 450	eBioscience	48-0451
CD335 (Nkp46)	29A1.4	Rat IgG2a, k	1:200	BUV737	BD Biosciences	565085
CD4	RM4-5	Rat IgG2a, k	1:400	PerCP-Cy5.5	Biolegend	100540
CD44	IM7	Rat IgG2b, k	1:400	PE-CF594	BD Biosciences	562464
CD49B	HMa2	Ham IgG	1:200	BUV395	BD Biosciences	740250
CD62L	MEL-14	Rat IgG2a, k	1:200	APC-Cy7	Biolegend	104428
CD8	53-6.7	Rat IgG2a, k	1:400	APC	Biolegend	100712
TCR-β chain	B20.6	Rat IgG2a, k	1:400	PE	Biolegend	127908
Live/Dead Fixable Green	-	-	1:800	FITC	Life Technologies	L23101
**Other antibodies**						
F4/80	BM8	Rat IgG2a, k	1:400	PE	eBioscience	12-4801
7AAD	-	-	1:400	-	Biolegend	420404
Trustain fcX	93	Rat IgG2a, k	1:400	-	Biolegend	101320

List of antibodies used for immunohistochemistry analysis and immunofluorescence analysis.

Antibody Specificity	Clone	Isotype	Dilution	Dye	Supplier	Catalog#
Ly6B.2	7/4	Rat IgG2a	1:7500	-	Bio-Rad	MCA771GA
F4/80	D2S9R	Rabbit IgG	1:6000	-	Cell Signaling	770765
CD4	4SM95	Rat IgG1, k	1:4000	-	eBioscience	14-976-32
CD8a	4SM15	Rat IgG2a, lambda	1:4000	-	eBioscience	14-0808-82
Cytokeratin 8/18		Rat IgG2a, k	1:100	-	University of Iowa Hybridoma Bank	TROMA-1
DAPI	-	-	5 μg/ml	-	Sigma	8417 – 10MG
