# Supplementary material for: Genetic programming of macrophages to perform anti-tumor functions using targeted mRNA nanocarriers
Source: Nat Commun. 2019 Sep 3;10:3974. doi: 10.1038/s41467-019-11911-5 (PMC6722139; doi:10.1038/s41467-019-11911-5)
Supplement: Supplementary file 2 — Reporting Summary [file 41467_2019_11911_MOESM2_ESM.pdf]

nature research

Corresponding author(s): Stephan, Matthias

Last updated by author(s): Aug 6, 2019

Reporting Summary

Nature Research wishes to improve the reproducibility of the work that we publish. This form provides structure for consistency and transparency in reporting. For further information on Nature Research policies, see Authors & Referees and the Editorial Policy Checklist.

Statistics

For all statistical analyses, confirm that the following items are present in the figure legend, table legend, main text, or Methods section.

n/a	Confirmed	
☐	☒	The exact sample size (*n*) for each experimental group/condition, given as a discrete number and unit of measurement
☐	☒	A statement on whether measurements were taken from distinct samples or whether the same sample was measured repeatedly
☐	☒	The statistical test(s) used AND whether they are one- or two-sided<br>*Only common tests should be described solely by name; describe more complex techniques in the Methods section.*
☐	☒	A description of all covariates tested
☒	☐	A description of any assumptions or corrections, such as tests of normality and adjustment for multiple comparisons
☐	☒	A full description of the statistical parameters including central tendency (e.g. means) or other basic estimates (e.g. regression coefficient) AND variation (e.g. standard deviation) or associated estimates of uncertainty (e.g. confidence intervals)
☐	☒	For null hypothesis testing, the test statistic (e.g. *F*, *t*, *r*) with confidence intervals, effect sizes, degrees of freedom and *P* value noted<br>*Give P values as exact values whenever suitable.*
☒	☐	For Bayesian analysis, information on the choice of priors and Markov chain Monte Carlo settings
☒	☐	For hierarchical and complex designs, identification of the appropriate level for tests and full reporting of outcomes
☐	☒	Estimates of effect sizes (e.g. Cohen's *d*, Pearson's *r*), indicating how they were calculated

Our web collection on statistics for biologists contains articles on many of the points above.

Software and code

Policy information about availability of computer code

Data collection	Bioluminescence was acquired using Living Image (R) version 4.3.1 software (Caliper Life Sciences). Flow cytometry data was acquired using FACSCanto software (FACSDiva v.6.1)
Data analysis	Bioluminescence was analyzed using Living Image (R) version 4.3.1 software (Caliper Life Sciences). Statistical analysis was done with Prism 7 software (Graphpad). Flow cytometry data was analyzed with FlowJo version 10.3.

For manuscripts utilizing custom algorithms or software that are central to the research but not yet described in published literature, software must be made available to editors/reviewers. We strongly encourage code deposition in a community repository (e.g. GitHub). See the Nature Research guidelines for submitting code & software for further information.

Data

Policy information about availability of data

All manuscripts must include a data availability statement. This statement should provide the following information, where applicable:

- Accession codes, unique identifiers, or web links for publicly available datasets
- A list of figures that have associated raw data
- A description of any restrictions on data availability

The Raw and processed data from the NanoString gene expression assays data have been deposited in the NCBI's Gene Expression Omnibus database under the accession code GSE120254 [https://www.ncbi.nlm.nih.gov/geo/query/acc.cgi?acc=GSE120254
] and GSE129498 [https://www.ncbi.nlm.nih.gov/geo/query/acc.cgi?acc=GSE129498
]. All the other data supporting the findings of this study are available within the article and its supplementary information files and directly from M. Stephan upon reasonable request. A reporting summary for this article is available as a Supplementary Information file.

Field-specific reporting

Please select the one below that is the best fit for your research. If you are not sure, read the appropriate sections before making your selection.

☒ Life sciences ☐ Behavioural & social sciences ☐ Ecological, evolutionary & environmental sciences

For a reference copy of the document with all sections, see nature.com/documents/nr-reporting-summary-flat.pdf

Life sciences study design

All studies must disclose on these points even when the disclosure is negative.

Sample size	We carried out our tumor studies in 10 mice per treatment group. This sample size provided us with 90% power to detect an effect size of 1 SD between groups, based on a one-way analysis of variance (ANOVA) with 2-sided significance level of 0.05 (calculated with Prism 6.0 Graph Pad software).
Data exclusions	All data were included in the analysis.
Replication	All experiments were replicated at least two times by two independent scientists working as a team on this project. Bioluminescent imaging and flow cytometry data were acquired and analyzed by two postdoctoral fellows working on this project
Randomization	Once tumors established, we randomized mice each into the control or treatment groups.
Blinding	Investigators were not blinded. All toxicity and safety studies (Figure 6, 7) were conducted by a board-certified staff pathologist in a double-blinded fashion

Reporting for specific materials, systems and methods

We require information from authors about some types of materials, experimental systems and methods used in many studies. Here, indicate whether each material, system or method listed is relevant to your study. If you are not sure if a list item applies to your research, read the appropriate section before selecting a response.

Materials & experimental systems

n/a	Involved in the study	
☐	☒	Antibodies
☐	☒	Eukaryotic cell lines
☐	☐	Palaeontology
☐	☒	Animals and other organisms
☒	☐	Human research participants
☒	☐	Clinical data

Methods

n/a	Involved in the study	
☒	☐	ChIP-seq
☐	☒	Flow cytometry
☒	☐	MRI-based neuroimaging

Antibodies

Antibodies used

CD45, clone 30-F11, Rat IgG2b, k, 1:800 dilution, eFluor 450, eBioscience cat# 48-0451
MHC I-A/I-E, clone M5/114.15.2, Rat IgG2b, k, 1:400 dilution, AlexaFluor700, Biolegend cat# 107622
CD11b, clone M1/70, Rat IgG2b, k, 1:200 dilution, APC, BD Biosciences cat# 557657
CD11C, clone N418, Arm Ham IgG, 1:200 dilution, PE-CF594, BD Biosciences cat# 562454
Ly6C, HK1.4, Rat IgG2a, k, 1:200, PerCP-Cy5.5, eBioscience, 45-4932
Ly6G, 1A8, Rat IgG2a, k, 1:200, APC-Cy7, Biolegend, 127624
CD38, 90/CD38, Rat IgG2a, k, 1:200, BUV395, BD Biosciences, 740245,
CD206, C0682C2, Rat IgG2a, k, 1:200, PE-Dazzle 594, Biolegend, 141732
Live/Dead Fixable Green, 1:800, FITC, Life Technologies, L23101
CD45, 30-F11, Rat IgG2b, k, 1:800, eFluor 450, eBioscience, 48-0451,
CD335 (Nkp46), 29A1.4, Rat IgG2a, k, 1:200, BUV737, BD 565085
CD4, RM4-5, Rat IgG2a, k, 1:400, PerCP-Cy5.5, Biolegend 100540
CD44, IM7, Rat IgG2b, k, 1:400 PE-CF594, BD 562464
CD49B,HMa2, Ham IgG, 1:200,BUV395, BD 740250
CD62L, MEL-14,Rat IgG2a, k, 1:200, APC-Cy7,Biolegend, 104428
CD8, 53-6.7, Rat IgG2a, k, 1:400, APC, Biolegend, 100712
TCR-beta chain, B20.6, Rat IgG2a, k, 1:400, PE, Biolegend 127908
F4/80, BM8, Rat IgG2a, k, 1:400, PE, eBioscience, 12-4801

Validation

Describe the validation of each primary antibody for the species and application, noting any validation statements on the manufacturer's website, relevant citations, antibody profiles in online databases, or data provided in the manuscript.

Eukaryotic cell lines

Policy information about cell lines

Cell line source(s)	Murine ovarian cancer cell line ID8 (provided by Dr. Katherine Roby, University of Kansas). THP1-Lucia ISG cells were purchased from InvivoGen (Cat# thp1-isg).
Authentication	This cell line was directly ordered from Invivogen
Mycoplasma contamination	These cell lines tested negative for Mycoplasma using the MycoAlertTM Mycoplasma Detection Kit (Lonza, Cat# LT07-118)
Commonly misidentified lines (See ICLAC register)	N/A

Palaeontology

Specimen provenance	N/A
Specimen deposition	N/A
Dating methods	N/A

☐ Tick this box to confirm that the raw and calibrated dates are available in the paper or in Supplementary Information.

Animals and other organisms

Policy information about studies involving animals; ARRIVE guidelines recommended for reporting animal research

Laboratory animals	Female Balb/C mice, female albino B6 (C57BL/6J-Tyr<c-2J) IACUC Assurance #A3226-01 (Fred Hutchinson Cancer Research Center)
Wild animals	N/A
Field-collected samples	N/A
Ethics oversight	The care and use of mice in this study was approved by the Institutional Animal Care & Use Committee (IACUC) at the Fred Hutchinson Cancer Research Center, and was in compliance with all relevant ethical regulations for animal testing and research (Assurance #A3226-01, IACUC Protocol Number 50782).

Note that full information on the approval of the study protocol must also be provided in the manuscript.

Flow Cytometry

Plots

Confirm that:

☒ The axis labels state the marker and fluorochrome used (e.g. CD4-FITC).

☒ The axis scales are clearly visible. Include numbers along axes only for bottom left plot of group (a 'group' is an analysis of identical markers).

☒ All plots are contour plots with outliers or pseudocolor plots.

☒ A numerical value for number of cells or percentage (with statistics) is provided.

Methodology

Sample preparation	Tissue was harvested and minced into 2-4 mm pieces using scissors and digested using Collagenase/Hyaluronidase (Stemcell, Catalog #07912) before sieving the cell suspension through a 100 µm cell strainer. Prior to antibody labeling, the cell suspension was incubated at 4 °C for 10 min with anti-murine CD16/CD32 FC-Receptor blocking reagent (final concentration of 2.5 µg/ml, dilution factor 1:200) to prevent unspecific binding.
Instrument	Data were acquired using a BD FacsCanto II cell analyzer
Software	FACSDIVA software (acquisition), FlowJo v10.1 (analysis)
Cell population abundance	The cell populations of interest were present in cell suspension at frequencies ranging from 0.1% to 40% (depending on the treatment group).
Gating strategy	ells were first gated for singlets (FSC-H vs. FSC- A) this gate was further analyzed for uptake of the Live/Dead Aqua stain to

Gating strategy	determine live versus dead cells and their expression of the relevant surface marker.

☒ Tick this box to confirm that a figure exemplifying the gating strategy is provided in the Supplementary Information.
